# In vivo evidence of microstructural hypo-connectivity of brain white matter in 22q11.2 deletion syndrome

**DOI:** 10.1038/s41380-023-02178-w

**Published:** 2023-07-26

**Authors:** Erika P. Raven, Jelle Veraart, Rogier A. Kievit, Sila Genc, Isobel L. Ward, Jessica Hall, Adam Cunningham, Joanne Doherty, Marianne B. M. van den Bree, Derek K. Jones

**Affiliations:** 1 Cardiff University Brain Research Imaging Centre (CUBRIC), School of Psychology, Cardiff University, Cardiff, UK; 2 Center for Biomedical Imaging, Department of Radiology, New York University Grossman School of Medicine, New York, NY, USA; 3 Medical Research Council Cognition and Brain Sciences Unit, University of Cambridge, Cambridge, UK; 4 Cognitive Neuroscience Department, Donders Institute for Brain, Cognition and Behavior, Radboud University Medical Center, Nijmegen, The Netherlands; 5 Neuroscience Advanced Clinical Imaging Service (NACIS), Department of Neurosurgery, The Royal Children’s Hospital, Parkville, VIC, Australia; 6 Centre for Neuropsychiatric Genetics and Genomics, Division of Psychological Medicine and Clinical Neurosciences, Cardiff University, Cardiff, UK; 7 Neuroscience and Mental Health Innovation Institute, Cardiff University, Cardiff, UK

## Abstract

22q11.2 deletion syndrome, or 22q11.2DS, is a genetic syndrome associated with high rates of schizophrenia and autism spectrum disorders, in addition to widespread structural and functional abnormalities throughout the brain. Experimental animal models have identified neuronal connectivity deficits, e.g., decreased axonal length and complexity of axonal branching, as a primary mechanism underlying atypical brain development in 22q11.2DS. However, it is still unclear whether deficits in axonal morphology can also be observed in people with 22q11.2DS. Here, we provide an unparalleled in vivo characterization of white matter microstructure in participants with 22q11.2DS (12–15 years) and those undergoing typical development (8–18 years) using a customized magnetic resonance imaging scanner which is sensitive to axonal morphology. A rich array of diffusion MRI metrics are extracted to present microstructural profiles of typical and atypical white matter development, and provide new evidence of connectivity differences in individuals with 22q11.2DS. A recent, large-scale consortium study of 22q11.2DS identified higher diffusion anisotropy and reduced overall diffusion mobility of water as hallmark microstructural alterations of white matter in individuals across a wide age range (6–52 years). We observed similar findings across the white matter tracts included in this study, in addition to identifying deficits in axonal morphology. This, in combination with reduced tract volume measurements, supports the hypothesis that abnormal microstructural connectivity in 22q11.2DS may be mediated by densely packed axons with disproportionately small diameters. Our findings provide insight into the in vivo white matter phenotype of 22q11.2DS, and promote the continued investigation of shared features in neurodevelopmental and psychiatric disorders.

## Introduction

22q11.2 deletion syndrome (22q11.2DS) is caused by a microdeletion of 1.5 to 3Mb on chromosome 22 [1]. It is the most common pathogenic copy number variant (CNV), with an estimated prevalence of 1 in 2000–6000 live births [2]. 22q11.2DS interferes with neurodevelopment across multiple systems, resulting in cardiac malformations, facial dysmorphology, immune deficiency and seizures/epilepsy, in addition to significantly enhanced risk of neurodevelopmental and psychiatric disorders, including learning disability, ASD, ADHD, developmental coordination disorder, and schizophrenia [3–5]. The occurrence of this syndrome has highly variable expressivity, but may offer a unique opportunity to characterize prominent features also present in individuals with complex, idiopathic conditions, such as schizophrenia [6].

Genetic, postmortem, and neuroimaging studies in schizophrenia patients have indicated that abnormal functional integration and disruptions in white matter structural connectivity are key features of neuropathology in this disease state [7–9]. However, schizophrenia shares symptoms and traits with many other neurodevelopmental and psychiatric disorders; thereby challenging a disease-specific phenotype that can be measured using in vivo technologies. 22q11.2DS has long been suggested as an important genetic and developmental model for schizophrenia as it affects up to 20–40% of individuals with 22q11.2DS [3]. By taking a genotype-first approach, new insights on how 22q11.2DS relates to prominent brain phenotypes are now possible even within a small number of individuals [10].

Neuronal connectivity has been identified as a key factor in the neurobiology of 22q11.2DS [11]. Using experimental animal models, disruptions in cellular energy production have been linked to neuronal deficits, such as decreased proliferation and altered morphology of axons, dendrites, and synapses [12, 13]. In turn, this leads to lower connectivity of long-range white matter tracts and impaired synchrony of neural activity [14]. The development of white matter for optimal brain communication requires the complex coordination of neuronal and glial cell types to fine-tune the timing and synchrony of action potentials throughout the brain [15]. This includes subtle variations in axonal morphometry and packing [16], myelination [17], synaptic densities and neurochemical mediators. Thus, a better understanding of atypical connectivity in white matter requires in depth assessment of factors that might contribute to trends observed in human studies.

Diffusion MRI (dMRI) is the only imaging modality that can explore both structural connectivity and microstructure of white matter tracts in vivo. DMRI reflects diffusion of water molecules on the micrometer length scale [18, 19]; thus, water acts as an in vivo probe, sensing obstacles constructed by cellular membranes and local fiber architecture, well below the spatial resolution of MRI [19, 20]. Most commonly, diffusion tensor imaging (DT-MRI) is used to parameterize the integrity or morphology white matter [21]. DT-MRI can be acquired in a relatively short scan time and has been broadly investigated across 22q11.2DS [22], typical development [23], and schizophrenia [9, 24]. The most commonly used metrics, fractional anisotropy (FA) and mean diffusivity $(\overset{-}{D}),$ have been associated with regional variations in microstructure, previously linked to myelination, axon density, and coherence. In particular, a recent, large-scale multi-site DT-MRI consortium study reported FA was higher in people with 22q11.2DS relative to age- and sex-matched controls for most WM regions, while measures of diffusivity were consistently lower [22]. This study provides a notable benchmark for research into 22q11.2DS; however, these differences were in contrast to previous observations in schizophrenia. Patients with schizophrenia have severe and widespread deficits in FA [9]. This creates a conundrum that both positive and negative alterations in FA could be associated with such direct overlap in symptoms and traits.

Conventional DT-MRI metrics are *sensitive* to a vast array of biophysical processes in tissue microstructure, but *specificity* is distinctly lacking [25]. This limitation can now be overcome by exploiting unusually high diffusion-weightings that have only recently become available through the development of customized ultra-strong MRI gradients for human neuroimaging [26, 27]. At such strong diffusion weightings (i.e., high *b*-values), the dMRI signal is dominated by contributions from water within cellular compartments such as axonal or glial processes [28]. Ultrastrong gradients are advancing the development of biophysical models of diffusion in tissue that hold the promise of quantifying important cellular features such as axonal diameters, densities and orientational dispersion in vivo [29, 30].

Here, we present an in-depth characterization of white matter microstructure in both typically-developing and 22q11.2DS participants using ultra-strong gradients and an extensive multishell dMRI acquisition, including *b*-values up to 6000 s/mm^2^. By so doing, we conducted a multiparametric analysis to gain novel insights into the microstructural properties underlying disrupted axonal morphology, to better describe previous observations of white matter hypo-connectivity in 22q11.2DS. The findings provide insight into 1) disentangling microstructural connectivity differences in brain white matter in 22q11.2DS versus typical developing participants, and 2) establishing the sensitivity and specificity of in vivo dMRI to underlying biophysical signatures of hypo-connectivity in white matter.

## Methods and Materials

### Subjects

This study included 92 typically-developing (TD) children (age range: 8–18 years, 49 F / 43 M) and 9 children with 22q11.2DS (age range: 11.8 to 15.2, 7 F / 2 M) (see Table 1 for full demographic information). From the TD group, a subset of children was selected based on age and sex (*n* = 22, age range: 12.0–15.5, 14 F / 8 M; age- and sex-matched group, ASM) for group comparison to children with 22q11.2DS. The Cardiff University School of Psychology Ethics Committee approved data collection procedures for the TD group, and each participant and/or their parent or legal guardian provided written informed consent. TD participants were recruited from the Cardiff area, and screened to exclude major neurological disorders, such as epilepsy. Participants with 22q11.2DS were recruited from the Experiences of CHildren with cOpy (ECHO) number variants study at Cardiff University (https://bit.ly/Cardiff-Echo-Study) [31]. ECHO study protocols were approved by NHS Wales Research Ethics Committee and brain imaging procedures were approved by the Cardiff University School of Medicine Ethics Committee. As part of the ECHO study, 22q11.2DS participants underwent extensive psychiatric, developmental and cognitive assessment including IQ assessment using the Weschler Abbreviated Scale Intelligence (WASI-II [32]). The ASM control group also performed an abbreviated 2-subtest WASI to establish a normative reference point in IQ scores. 22q11.2DS also provided information about social communication difficulties indicative of ASD from the Social Communication Questionnaire (SCQ [33]), and psychiatric interviews were taken using the Child and Adolescent Psychiatric Assessment (CAPA [34]). Seizure and medication history were obtained from parental questionnaires [35]. One child had a history of febrile seizures, but no other seizure history or psychiatric/ epilepsy medications were reported. All participants underwent the same pre-scan procedure, which included an in-person screening for MRI safety and a practice scan in a mock MRI scanner.

### Image acquisition and preprocessing

All imaging data were acquired on a 3 T Connectom scanner (Siemens Healthcare, Erlangen, Germany) with 300 mT/m gradients and a 32-channel radiofrequency coil (Nova Medical, Wilmington, MA, USA) at the Cardiff University Brain Research Imaging Centre (CUBRIC).

T1-weighted anatomical images were acquired using a 3D Magnetization Prepared Rapid Gradient Echo (MP-RAGE) sequence with the following parameters: echo time of 2 ms, repetition time of 2300 ms, time to inversion of 857 ms, and flip angle of 9°. The field of view was 256 × 256, the imaging matrix 256 × 256, with 192 slices of 1mm slice thickness. The total scan time was 5 min and 32 s.

dMRI data were acquired using a multi-shell diffusion-weighted EPI sequence. Images covered the whole brain with field of view 220 × 220 mm, imaging matrix 110 × 110, and 66 slices of 2mm slice thickness. dMRI were acquired using the Stejskal-Tanner sequence with an anterior-to-posterior phase-encoding direction, with an echo time of 59 ms, repetition time of 3000 ms, and with diffusion-weighting gradient separation Δ = 23.3 ms and duration *δ*= 7 ms. Data were acquired at five different *b*-values: *b* = 500, 1200, 2400, 4000, and 6000 s/mm^2^; with 30 and 60 non-collinear directions used for *b* ≤ 1200 s/mm^2^ and *b* ≥ 2400 s/mm^2^, respectively [36]. At the maximum diffusion weighting, the gradient amplitude was 285.6 mT/m. In addition, 14 non-diffusion weighted images were distributed uniformly throughout the protocol. One additional volume was acquired with reversed phase encoding for the purpose of EPI distortion correction. The total dMRI scan time was 16 min and 14 s.

The following preprocessing steps were used to reduce thermal noise and image artifacts: image denoising [37], correction for signal drift [38], motion, eddy current, and susceptibility-induced distortion correction [39], gradient non-linearities, Gibbs ringing [40], and outlier rejection [41]. The preprocessing pipeline was implemented in MATLAB (The MathWorks, Natwick, MI, USA), and depended on open source software packages from MRtrix [42] and FSL [43].

### Quantitative diffusion metrics

There has been little consensus as to which dMRI metric provides the most sensitive or specific characterization of the biophysical properties of white matter. In this work, we use an inclusive approach rather than focus on a single analysis strategy, examining a large number of distinct models across the feature space of dMRI (including low, intermediate and high b-value regimes). These include (i) DT-MRI, (ii) diffusion kurtosis imaging (DK-MRI), (iii) the rotationally invariant spherical moments of the signal at various *b*-values (i.e., spherical harmonics), and (iv) the Biophysical Standard Model (BSM) [29]. A comprehensive overview of the individual metrics that are derived from those models and used in this study is provided in Suppl. Section: *Descriptions of quantitative diffusion metrics*. In brief, we will refer to the following metrics. DT-MRI includes fractional anisotropy (FA), mean diffusivity $(\overset{-}{D}),$ radial diffusivity (D_⊥_), and axial diffusivity (D_∥_). DK-MRI includes mean kurtosis $(\overset{-}{\text{K}}),$ radial kurtosis (K_⊥_), and axial kurtosis (K_∥_). The spherical harmonics include spherical mean at low-*b* (*Ṡ_μ_*(*b* = 500), spherical mean at high-*b* (*Ṡ_μ_*(*b* = 6000)), spherical variance at low-*b* (*Ṡ_σ_*(*b* = 500), spherical variance at high-*b*, (*Ṡ_σ_*(*b* = 6000)). The BSM includes the intracellular signal fraction (*f*), parallel intracellular diffusivity (*D_c_*), parallel and perpendicular extracellular diffusivity $\left(D_{e}^{∥},D_{e}^{⊥}\right)$ and orientational alignment (*κ*). In addition, the precision of individual metrics is evaluated in a simulation experiment; see Suppl. Section *Precision of diffusion metrics*.

### Unique sensitivity to axon diameter

The Connectom 3 T MRI scanner is a unique research tool with only four sites available worldwide. This scanner allowed for experimental set up that is sensitive to axon morphology, such as diameter; see Suppl. Section *Sensitivity to axon morphology*. The combination of strong gradients (300 mT/m versus 40–80 mT/m in clinical scanners) and short diffusion gradient duration yields an unprecedented sensitive to the inner axon diameter. Unfortunately, the direct measurement of axon diameters using dMRI remains challenging due to long scan times that might be unattainable in vulnerable cohorts. However, for the first time, the axon diameter is encoded in the dMRI data measured at high *b*-values due to the use of ultra-strong gradients.

### Biophysical principal components

dMRI metrics are sensitive to a wide array of microscopic components, such as axonal density and packing, intra-voxel fiber orientational dispersion, membrane permeability, and myelination; however, a single metric on its own, such as FA, lacks specificity to any one particular feature of the white matter microstructure. Additional information is needed to assign more specific interpretation, but acquiring a large volume of complex dMRI data becomes difficult to analyze. To overcome this, we applied a dimensionality reduction technique to uncover hidden structures, or latent variables, that aggregate shared biophysical features across the observed data allowing for more specific interpretation without the statistical limitations imposed by a biophysical model. This approach is particularly powerful as it is not hindered by the potential correlation between metrics or redundancy that is also present in dMRI.

A principal component analysis (PCA) was used to identify latent structure in total white matter estimates [44]. The full TD cohort (*n* = 92) was used to train the model. The data sampling adequacy was assessed using a Kaiser-Meyer-Olkin (KMO) test and the Bartlett’s test of sphericity. Parallel analysis was used to select the optimal number of principal components. We used an oblique matrix rotation to allow underlying biophysical components to correlate, as this is likely most biologically plausible. The loadings per principal component were used to compute a composite total, or PCA component score.

### Segmentation of the white matter and individual tracts

Digital brain extraction was performed using the FSL Brain Extraction Tool (BET) [43] followed by the FSL segmentation tool (FAST) [45] in which three tissue types, i.e., CSF, WM, and GM were segmented. Moreover, automated tract segmentation software (TractSeg) was used to extract eleven white matter fiber tracts using the *b* = 6000 s/mm^2^ shell for each participant [46]. Tract selection was referenced from previous developmental neuroimaging studies using dMRI [23]. For this study, these include association tracts: arcuate fasciculus (AF), cingulum (CG), inferior fronto-occipital fasciculus (IFO), inferior longitudinal fasciculus (ILF), superior longitudinal fasciculus (SLF), and uncinate fasciculus (UF); commissural tracts: genu (GCC), body (BCC), and splenium (SCC) of the corpus callosum; and projection tracts: corticospinal tract (CST), optic radiation (OR). All tract reconstructions were bilateral except for the inter-hemispheric projections of the corpus callosum; see Suppl. Fig. 1.

### Tract volume quantification

The volume of each segmented fiber pathway was estimated by creating a tract density map using the associated streamlines [47], then counting the total number of voxels in which streamlines were observed. Nominal tract volumes were normalized for total brain volumes to compensate for brain size differences. Since each individual streamline is strongly susceptible to thermal noise, the estimation of volume quantification might be less accurate for fiber pathways with high surface-to-volume ratio. For our cohort, the minimally detectable effect size is 10 to 20% (cf. Suppl. Section *Tract Volume Quantification*, Suppl. Fig. 2). Therefore, the sensitivity of the technique is intrinsically limited to relatively large changes in typical and/ or atypical development.

### Along tract profiling

The estimation of the BSM parameters on a voxel level is challenging because of the poor precision and degeneracy in the model at such a low signal-to-noise ratio (SNR), i.e., multiple solutions are possible for one signal [48]. However, by adopting a novel approach of averaging the (rotationally-invariant) spherical moments within tract segments prior to model fitting, we were able to boost the SNR of our data and thus the reliability of BSM parameter estimates, thereby fostering more accurate and precise biophysical modeling. Note, the curvature of tracts does not bias the model parameters because they are derived from the rotationally invariant spherical moments of the signal.

Specifically, we projected the rotationally-invariant spherical moments of each individual tract onto twenty equidistantly-spaced nodes of the associated centerline. This in-house strategy was implemented in MATLAB, inspired by tractometry methods such as the AFQ toolbox and others [49, 50]. Each centerline was computed by fitting a threedimensional spline through equidistantly-spaced sample points of 10,000 streamlines that constituted an individual bundle [49]. The intrinsic challenges of fiber tracking, i.e., spurious connections, might propagate in this analysis [51]. Therefore, quality control was performed using visual inspection of centerline geometry and positioning. From this, we identified the body of the corpus callosum, or BCC, to be poorly robust due to streamlines occurring in spurious directions. The BCC was removed from subsequent comparisons of 22q11.2DS participants. In addition, along-tract metric profiles for all subjects were aligned to account for varying tract lengths and head sizes using a linear translation and rescaling of the nodes.

### Sensitivity to age-effects in typical development

Age-effects of dMRI are well reported in the literature and we use this analysis to establish the sensitivity of the PCA technique. The associations between age and PCA scores/individual metrics within selected tracts were assessed using the goodness-of-fit for both linear (age) and quadratic (age^2^) models as reported in the literature for developmental cohorts [23]. Using the corrected Akaike information criterion (AIC) to compare linear versus quadratic models, we found no statistical evidence to motivate the more complex (i.e., quadratic) model for this age range. Therefore, all age-associations for PCA scores/individual metrics in total white matter and tracts were assessed using a simple linear regression model for age. Model statistics for each comparison with age are reported as the *r*^2^ and unstandardized *β* coefficient. The statistical significance level (*p* ≤ 0.035) is determined using the Benjamini–Hochberg procedure to control the false discovery rate (FDR) in this study [52]. Predicted metric scores were computed using the regression equation for youngest (8 year old) and oldest (18 year old) participants. The difference in predicted metric scores was used to illustrate age-effects in the TD group.

### Group differences in 22q11.2DS and age- and sex-matched controls

Standardized effect size was computed between age- and sex-matched controls (ASM) and 22q11.2DS participants. Here, we report Hedge’s *g*, which corrects for bias that might be present due to small sample sizes [53]. The statistical significance level is *p* ≤ 0.011 after FDR correction. The *t*-statistic and degrees of freedom *df*(*N*-1) are reported for independent group comparisons. Feature extraction and statistical analyses were conducted using R v3.4.1 (Single Candle) [54] and MATLAB.

## Results

### Multidimensional in vivo dMRI captures biophysical components of white matter

**Fig. 1:**
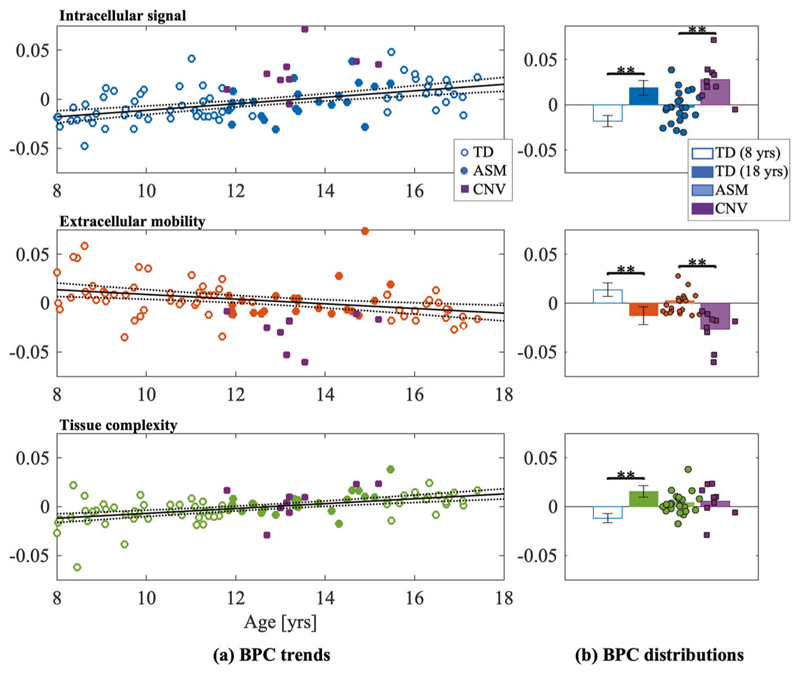
Age associations with the three biophysical principal components: intracellular signal (black), extracellular mobility (red), and tissue complexity (green). **a** BPC values are correlated with age in total white matter. Points are highlighted for age- and sex-matched controls (filled circles), and 22q11.2DS participants (filled squares). The black line denotes the linear regression for the TD group only (with dashed 95% confidence intervals). **b** Nominal values are computed for 1) estimates from the linear regression for youngest (8 year old) and oldest (18 year old) TD participants, and 2) measured BPC scores of ASM and 22q11.2DS participants. Asterisk denotes significance between group means (*p < 0.05, **p < 0.001).

A PCA analysis was performed to characterize latent features present in dMRI data of white matter microstructure, Fig. 1. Parallel analysis in total white matter suggested that three components (dubbed here ’Biophysical Principal Components’ or BPCs) derived from eleven dMRI metrics were adequate to explain the data, together explaining 91% of the total variance in white matter. Parallel analysis was also applied to individual tracts, again confirming three components with the same groups of variables. Data sampling adequacy was verified by the KMO-statistic, KMO = 0.68, and Bartlett’s test of sphericity, *x*(55) = 2005, *p* < 0.05. Table 2 shows the standardized component loadings of each metric after oblique rotation.

**Fig. 2:**
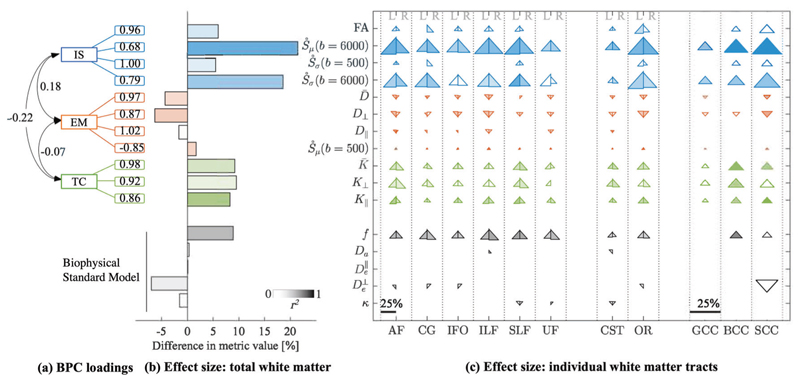
dMRI fingerprint of development. Metrics are grouped by intracellular signal (IS, blue), extracellular mobility (EM, red), tissue complexity (TC, green), and Biophysical Standard Model parameters (black). **a** The highest metric loadings are shown for each BPC. **b** Bar plots are encoded with linear regression statistics in total white matter. Color intensity is effect size (*r*^2^); bar length is percent difference of metric estimates from the linear regression for youngest (8 year old) and oldest (18 year old) TD participants. **c** Triangle plots are the same statistics for individual white matter tracts. Triangles are divided by left (L) and right (R) hemisphere for association and projection tracts. Commissural tracts span both hemispheres and are not divided. Percent differences are denoted by the scale bar, reaching a maximum of 25% difference. Triangle orientation, i.e., up or down, denotes positive or negative associations with age, respectively. Triangles are omitted that do not survive statistical significance.

Inspection of the loading matrix showed that each of the three BPC’s could be described as capturing a specific biophysical dimension of microstructural features in white matter, as indicated by high component loadings for groups of variables (>0.5; Fig. 2). Here, we introduce labels to represent the most salient and unifying properties from the individual, observable metrics that contribute to each component. These are: intracellular signal, extracellular mobility, and tissue complexity.

The first component, intracellular signal, captured 31% of the variance in the data, and was characterized most strongly by FA, *Ṡ_μ_*(*b* = 6000), *Ṡ_σ_*(*b* = 500), *Ṡ_σ_*(*b* = 6000). FA is generally non-specific and has been associated with many tissue properties [25]; however, when combined with high-b (*Ṡ_μ_*(*b* = 6000), *Ṡ_σ_*(*b* = 6000)) and *Ṡ_σ_*(*b* = 500), the resultant feature is more selective for intracellular signal. This is in part, due to diffusion weightings at high *b*-values (*b* = 6000 s/mm^2^) acting to suppress signals from the extracellular matrix. An in-depth technical discussion on interplay of spherical harmonic metrics and FA is provided in Suppl. Section *The biophysical interpretation of the intracellular signal component*.

The second component, extracellular mobility, captured 33.1% of variance in the data, with high loadings for $\overset{-}{D},$ *D*_⊥_, *D*_∥_, and *Ṡ_μ_*(*b* = 500). These metrics exist in the low-*b* regime (e.g., *b* < 2000 s/mm^2^), in which extra- and intra-cellular signal contribute to the signal.

And finally, the third component, tissue complexity, captured 27.7% variance in the data, with highest loadings for $\overset{-}{K},$ *K*_⊥_, *K*_∥_. Kurtosis metrics have previously been used to assess tissue complexity within the white matter microenvironment. Kurtosis is quadratically more sensitive than DT-MRI and therefore a powerful tool in detecting subtle differences [55].

### BPCs are sensitive to age effects

Regression scores showed sensitivity to age for all BPCs; however, the strongest association with age was found to be tissue complexity (*r*^2^ = 0.369, *β* = 0.002, *p* < 0.0001). This was closely followed by intracellular signal (*r*^2^ = 0.325, *β* = 0.003, *p* < 0.0001), and extracellular mobility (*r*^2^ = 0.222, *β* = -0.002, *p* < 0.0001) (Fig. 1a).

In total white matter, metrics associated with intracellular * o signal, i.e., *Ṡ_μ_* and *Ṡ_σ_* at high *b*-values, had the largest differences versus all other metrics in predicted values between ages 8 and 18 of ~20%. This large effect was reflected by strong associations with age (*Ṡ_μ_*(*b* = 6000): *r*^2^ = 0.553, *β* = 0.002, *p* < 0.0001 ; and *Ṡ_σ_*(*b* = 6000): *r*^2^ = 0.369, *β* = 0.0002, *p* < 0.0001) (Fig. 2). Significant age-associations were also found at the tract level for both metrics, as shown in Fig. 2c. In comparison, total white matter *Ṡ_σ_* at low *b*-value and FA were less sensitive to age effects (*Ṡ_σ_*(*b* = 500): *r*^2^ = 0.120, *β* = 0.0001, *p* = 0.001; and FA: *r*^2^ = 0.165, *β* = 0.002, *p* = 0.0002) and had negligible effects at the individual tract level.

For extracellular mobility metrics, $\overset{-}{D},$ *D*_⊥_, and *Ṡ_μ_*(*b* = 500) had significant associations with age in total white matter $(\overset{-}{D}:$ *r*^2^ = 0.216, *β* = −0.004, *p* < 0.0001; *D*_⊥_: *r*^2^ = 0.294, *β* = −0.005, *p* < 0.0001; *Ṡ_μ_*(*b* = 500): *r*^2^ = 0.248, *β* = 0.001, *p* < 0.0001). These associations were also present for most tracts. *D*_∥_ did not have any notable associations with age for total white matter or individual tracts.

Tissue complexity metrics had high sensitivity to age for total white matter and most individual tracts. In total white matter, *K*_∥_ had the strongest association (*K*_∥_: *r*^2^ = 0.55, *β* = 0.005, *p* < 0.0001), followed by $\overset{-}{K}$ (*r*^2^ = 0.28, *β* = 0.007, *p* < 0.0001) and *K*_⊥_ (*r*^2^ = 0.16, *β* = 0.008, *p* = 0.0002).

### Participants with 22q11.2DS have higher intracellular signal and lower extracellular mobility compared to age- and sex- matched controls

Intracellular signal fraction was increased relative to ASM controls (*t*(29) = 4.136, *p* < 0.001, *g* = 1.594), and lower extracellular mobility (*t*(29) = − 3.897, *p* < 0.001, *g* = 1.502) (Fig. 1b). There were no differences in tissue complexity (*t*(29) = 0.355, *p* = 0.725, *g* = 0.137).

**Fig. 3:**
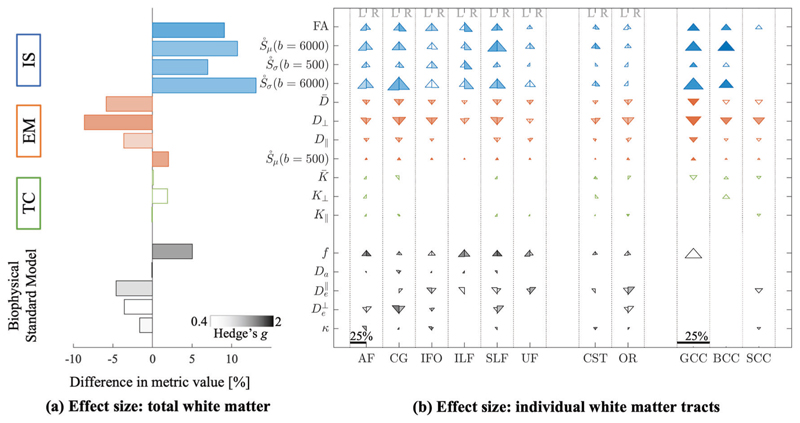
dMRI fingerprint of 22q11.2DS. Metrics are grouped by intracellular signal (IS, blue), extracellular mobility (EM, red), tissue complexity (TC, green), and Biophysical Standard Model parameters (black). **a** Bar plots are encoded by mean difference and effect size in total white matter. Color intensity is effect size (Hedge’s *g*); bar length is percent difference between average metric values for ASM and 22q11.2DS participants. **b** Triangle plots are the same statistics for individual white matter tracts. Triangles are divided by left (L) and right (R) hemisphere for association and projection tracts. Commissural tracts span both hemispheres and are not divided. Percent differences are denoted by the scale bar, reaching a maximum of 25% difference. Triangles pointed up indicate a higher metric values for 22q11.2DS relative to ASM (and vice versa for down). Data from BCC is not shown for the Biophysical Standard Model parameters due to lack of robustness in the along-tract profiling for that particular tract (marked as –). Triangles are omitted for low and moderate effect sizes, Hedge’s *g* < 0.4, and left blank if not significant, *p* < 0.011.

A summary of individual quantitative diffusion metrics in total white matter and tracts is found in Fig. 3. In total white matter, intracellular signal was led by FA (*t*(29) = 4.611, *p* < 0.001, *g* = 1.777), followed by *Ṡ_σ_*(*b* = 500), (*t*(29) = 3.793, *p* < 0.001, *g* = 1.462); *Ṡ_σ_*(*b* = 6000), (*t*(29) = 3.789, *p* < 0.001, *g* = 1.460); and finally *Ṡ_μ_*(*b* = 6000), (*t*(29) = 3.673, *p* = 0.001, *g* = 1.416).

22q11.2DS had higher individual metric values of intracellular signal relative to ASM controls for most individual tract types. Only the low-b metric, *Ṡ_σ_*(*b* = 500) had consistently smaller or absent significant effects across tracts.

Extracellular mobility was decreased in 22q11.2DS compared to ASM for total white matter. The highest effect was in *D*_⊥_ (*t*(29) = − 4.450, *p* < 0.001, *g* = 1.715), followed by *Ṡ_μ_*(*b* = 500) (*t*(29) = 4.205, *p* <0.001, *g* = 1.620), and $\overset{-}{D}$ (*t*(29) = − 3.660, *p* < 0.001, *g* = 1.411). Only *D*_∥_ did not reach significance threshold with the FDR correction (*t*(29) = 0.992, *p* < 0.015, *g* = 0.992).

At the tract level, the diffusivity metric, *D*_⊥_ was most consistently different, being significantly less for all tracts except UF. $\overset{-}{D}$ was also consistent, with significant effects for all tracts except ILF, CST (L), BCC, and SCC. Effects remained small or absent for *D*_∥_ as seen in total white matter. For *Ṡ_μ_*(*b* = 500), effects were relatively small for most tracts (here showing an increase, which corresponds to decreased diffusivity).

Tissue complexity metrics, $\overset{-}{K},$ *K*_⊥_, *K*_∥_, were not sensitive to group differences for total WM or at the tract level. This is a notable omission, as kurtosis metrics were the most sensitive to age effects in the TD group.

### Biophysical modelling in support of intracellular signal

For the TD cohort, intracellular signal fraction (*f*) showed the greatest sensitivity to age in total white matter (*r*^2^ = 0.299, *β* = 0.005, *p* < 0.0001), being ~ 9% higher over the age range. These effects were significant for most tracts with percent differences ranging from ~ 3 to 16%. The $D_{e}^{⊥}$ and *κ* were lower with age in total white matter $(D_{e}^{⊥}:$ *r*^2^ = 0.094, *β* = −0.005, *p* = 0.005; *κ*: *r*^2^ = 0.019, *β* = −0.0007, *p* = 0.11), although *κ* did not reach significance here. *D_c_* and $D_{e}^{∥}$ were not sensitive to age effects.

Participants with 22q11.2DS had ~ 5% higher *f*, ~ 4.6% lower $D_{e}^{⊥}\sim 3.6\%$ lower $D_{e}^{∥},$ and ~ 1.6% lower *κ* versus the ASM control group. Only *f* survived the statistical threshold (*t*(29) = 3.474, *p* = 0.0016, *g* = 1.339). The remaining metrics were not sensitive to differences, possibly reflecting poor precision inherent in fitting a large number of model parameters (Suppl. Fig. 3). At the individual tract level, *f*, $D_{e}^{⊥},\;D_{e}^{∥}$ had some sensitivity to group differences of primarily association tracts. While biophysical modelling holds the promise of more specific interpretation, it is challenged by lower precision in parameter estimates. In Suppl. Fig. 3, the precision of metrics and BSM model parameters is shown, highlighting this discrepancy, e.g., *D_c_* and $D_{e}^{∥}.$ Therefore, the combination of both sensitive and specific dMRI metrics expands our inferences to allow for in-depth phenotyping of white matter microstructure.

### Participants with 22q11.2DS have lower total WM and individual tract volumes compared to age- and sex-matched controls

**Fig. 4:**
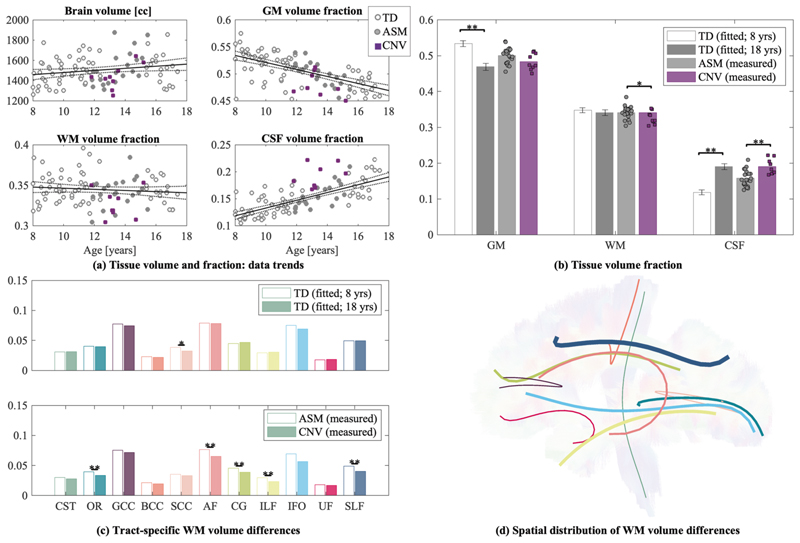
Whole brain and tract volume fractions in TD and 22q11.2DS participants. **a** Total brain volume and gray matter (GM), white matter (WM), and cerebrospinal fluid (CSF) volume fractions are correlated with age. Points are highlighted for ASM (filled circles) and 22q11.2DS participants (filled squares). The black line denotes the linear regression line for the TD group only (with dashed 95% confidence intervals). **b** Nominal differences in GM, WM, and CSF are shown for volume fraction estimates from the linear regression for youngest (8 year old) and oldest (18 year old) TD participants. In addition, measured volume fractions are shown for ASM and 22q11.2DS participants (data points overlaid on bar). **c** Individual WM tract volume fractions are shown for (top) youngest (8 year old) and oldest (18 year old) TD, and (bottom) ASM and 22q11.2DS participants. **d** Percent differences in WM tract volume fractions for ASM and 22q11.2DS participants are overlaid on a single subject’s tractogram. The radius of each tract ‘tube’ reflects the percent difference between groups (ranging from 0–23%). Tract colors match panel C. Asterisk denotes significance between groups (*p < 0.05, **p < 0.001).

Whole brain and individual tract volumes were computed for both TD and 22q11.2DS groups, see Fig. 4. For the TD group, there were significant age effects for lower GM volume and higher CSF volume with age (GM: *r*^2^ = 0.536, *β* = −0.007, *p* < 0.0001; CSF: *r*^2^ = 0.579, *β* = 0.007, *p* < 0.0001), aligning with previous developmental literature [56, 57]. There were no significant age effects for total brain volume or WM volume which is not consistent with other findings. In addition, the WM volume analysis per tract was not sensitive to age effects.

Compared to ASM, 22q11.2DS children had significantly higher CSF volume (*t*(29) = 3.705, *p* < 0.001, *g* = 1.428) and lower WM volumes (*t*(29) = − 2.197, *p* = 0.036, *g* = 0.847). There were no differences in whole brain or GM volumes (Fig. 4a, b). As shown in Fig. 4c, there were significant reductions in most individual tract volumes for 22q11.2DS. Differences in tract volumes were large, ranging from 9 − 28%, and were present irrespective of tract type or location. Figure 4d highlights the global pattern of these differences across the brain.

## Discussion

### Replicability of previous studies

22q11.2DS has been previously studied using DT-MRI metrics for almost 20 years. The landscape of these studies is complex, resulting from differences in study design, inclusion criteria and sample sizes, clinical hardware (1.5 T to 3 T), and analysis approach. Most recently, the ENIGMA-22q11.2DS working group harmonized data across 10 study sites in the largest DT-MRI study ever completed in this population [22]. This work included 594 participants: 334 with 22q11.2DS and 260 age- and sex-matched controls (age range: 6–52 years) and serves as the most comprehensive benchmark data set for the evaluation of WM differences in 22q11.2DS.

Overall, the ENIGMA results highlight the presence of a global WM phenotype in 22q11.2DS. For this study, similar patterns of increased FA for most tracts and decreased diffusivity metrics for $\overset{-}{D},$ *D*_⊥_, *D*_∥_ were observed in 22q11.2DS relative to controls. The main exceptions were three association regions - the SLF, external capsule, and fornix where the ENIGMA authors observed significantly lower FA. We did not include external capsule and fornix, however, this study did not detect lower FA in SLF, Fig. 3. This could be due to a number of differences in study design and/or methodology. For example, the age range of the ENIGMA study was much broader. White matter development is heterochronistic, meaning different regions develop at different times and rates. In this way, association tracts, such as SLF, have the most protracted development, continuing into the third decade of life and beyond [17, 23]. It is possible that competing maturational processes such as ongoing myelination may confound inferences over a broad age range, leading to the observed differences. A second possibility is that the ROIs used in the ENIGMA study do not directly correspond to the tractography approach used here. This is discussed in Suppl. Section, *Comparison to ENIGMA DT-MRI results*, alongside an in-depth comparison of DT-MRI metrics between these studies (Suppl. Table 1).

### In-depth microstructural phenotyping of white matter in 22q11.2DS

The dMRI signal reflects a diverse array of microstructural attributes, including axonal density and packing, intra-voxel fiber orientational dispersion, membrane permeability, and myelination. Therefore, it is an inherent challenge to extract inferences on which specific process, or combination of processes, is most likely contributing to dMRI metrics. The utility of PCA techniques for grouping MRI measures in biophysically relevant ways has been demonstrated previously [44, 58, 59]. Here, this approach allowed us to probe the sensitivity of dMRI to differences in 22q11.2DS while characterizing three distinct sources, or BPCs, representative of the underlying microstructure. Accepting the risk of oversimplification, we coined these BPCs as “intracellular signal”, “extracellular mobility”, and “tissue complexity”.

By comparing the 22q11.DS to age- and sex-matched matched controls, projecting the differences onto trends of typical development in adolescence, we conclude that children with 22q11.2DS had dMRI profiles most similar to older children. This holds for intracellular signal and extracellular mobility, in addition to their composite metrics for both total white matter and individual tracts. Tissue complexity was not sensitive to group differences. This component, comprised exclusively of DKI metrics, was in contrast to strong developmental effects observed in the TD group.

### Volumetric differences in WM

In addition to the diffusion metrics quantifying microstructure, we observed significant morphological differences in total WM and white matter bundles. Specifically, we identified lower white matter volume in the following tracts for 22q11.2DS: AF, CG, ILF, SLF, and OR. As seen in Fig. 4d, these tracts represent widespread decreases throughout the brain, which are also in agreement with previous studies of 22q11.2DS [60]. We did not observe significant age-related differences in WM volume across the age-span studied here in the typically developing cohort. However, the reduction in GM volume and higher CSF volume is consistent with the TD literature [57]. In what follows, we will argue that the combination of changes in dMRI metrics that were sensitized to axonal morphology at the *micro*-scale, and lower white matter volume in the 22q11.2DS participants at the *macro*-scale, provides evidence in support of the hypo-connectivity hypothesis in 22q11.2DS and a lower number of large axons in particular.

### In support of hypo-connectivity

To examine the biological plausibility of our findings, we can look at converging evidence from experimental animal models of 22q11.2DS. For instance, Fernandez et al. characterized under-connectivity as limited axon and dendrite growth, and disrupted mitochondria and synaptic integrity [12]. These effects were specific to pyramidal neurons in layer 2/3 of the cortex, which primarily support long-distance cortico-cortico connections. In a separate mouse model used to report on genetic susceptibility of schizophrenia, altered axonal growth, branching, and disrupted connectivity were linked to behavioral deficits [13, 14]. In these studies, both short- and long-range connectivity was impacted. The size of terminal axonal branching correlates with the caliber of axons - therefore, large diameter axons are capable of high rates of information transfer at these junctions [61–63]. Axonal growth and branching are energetically demanding processes. Therefore, in 22q11.2DS, where mitochondrial dysfunction has been linked to atypical axonal morphology [12], there may be a critical susceptibility for the large neurons. To date, we are not aware of any studies measuring axon diameter distributions specifically in 22q11.2DS; however, studies of other neurodevelopmental disorders, such as ASD and Angelman Syndrome, have reported a lower number of large axons in comparison to typical development [64–66]. Postmortem tissue from schizophrenia subjects also shows specificity to alterations in neuron morphology, such as dendritic spine loss [67]. Therefore, we hypothesize that the lower white matter tract volumes in 22q11.2DS are representative of densely packed axons with disproportionately small diameters.

The 22q11.2 deletion is highly penetrant in affected individuals but leads to heterogeneous risk profiles and presentations. Recent work on the genetic basis of 22q11.2DS has suggested there may be a consortium of gene effects - outside the 22q11.2 locus itself - that contribute to the onset of schizophrenia or ASD [68]. In addition, environmental factors may lead to a stochastic cascade of phenotypic variation over the lifespan (reviewed in [69]). Ultimately, many factors must be considered when evaluating the expression of phenotypes related to 22q11.2DS. As this body of work continues to grow, a more in-depth characterization of established brain phenotypes can provide new insights underlying potential mechanisms of WM hypoconnectivity [10].

### Sensitivity to axonal morphology

A unique feature of our study is the sensitivity to axonal morphology at *b* = 6000 s/mm^2^ achieved by exploiting the ultrastrong gradients, i.e., 300mT/m, of the Connectom scanner. Our data did not allow for the direct quantification of the axon diameter (as discussed below in *Considerations for scanning*). However, various metrics are sensitive to the axon diameter and are, as such, directly sensitive to differences in the axon diameter distribution, with particular sensitivity to differences in the number and diameter of large axons [70]. The key parameters are the spherical mean and variance at the highest *b*-value. The significant increase in both parameters in the 22q11.2DS compared to age- and sex-matched controls is consistent with a reduction in mean axon diameter. Of relevance to our observation of higher FA in 22q11.2DS and in line with the ENIGMA-22q11.2DS DT-MRI study [22], an association between higher FA (and smaller *D*_⊥_) and smaller axons has previously been demonstrated [71].

Moreover, there is an inverse relationship between axon diameter and axon density, i.e., larger diameter axons take up more space, due not only to larger size, but also from the space demands of neighboring glial cells such as myelinating oligodentrocytes and astrocytes [72–74]. When there are fewer large diameter axons, or their morphology is less complex due to fewer branch points, the space requirements go down - in agreement with the significant loss of tract-based white matter volumes seen in our study. As a consequence, the *relative* proportion of smaller diameter axons increases, leading to greater axonal density and the reduced $D_{e}^{⊥}.$ Moreover, dMRI has a reduced (or even nullified) sensitivity to smaller axons as a result of which the dMRI signal is less attenuated (see Suppl. Fig. 4). Both effects are consistent with higher values of *Ṡ_μ_* and *f* as seen in 22q11.2DS relative to ASM controls. Finally, higher orientational dispersion, (i.e., drop in orientational-concentration parameter *κ*), in combination with lower axial diffusivity, might reflect a more tortuous path of axons that has been associated with smaller axons [75, 76]. This hypothesis was also put forward by the ENIGMA working group based on the lower axial diffusivities in their study [22]. However, a more direct measurement of tortuosity along segments of axons depends on *D_c_*, a metric that was not estimated with sufficient precision using the Biophysical Standard Model to support this hypothesis.

### Typical development

Although this work focuses on 22q11.2DS, our results also provide new insights into typical development. There is ongoing debate about the biophysical underpinnings of brain maturation: axonal growth versus myelination [77]. To this end, the extended multishell dMRI data, including the high *b*-values, complement previous observations of enhanced sensitivity to age effects (e.g., *b* = 3000) [78, 79]. Notably, from the high *b*-value data, *Ṡ_μ_* and *Ṡ_σ_* are the most sensitive parameters amongst all evaluated metrics. Given that the signal from the extra-cellular space is effectively filtered out at these b-values, this result suggests that the dynamics of the neuronal and/ or glial processes, rather than the extra-cellular signal, are dominating effects related to age-dependency in dMRI. As suggested by [77], white matter maturation might encompass axonal growth or swelling due to neuronal activity [80, 81] or even hormonal fluctuations [82, 83]. However, the increase in axon diameters exclusively would have resulted in a lower *Ṡ_μ_*(*b* = 6000) (see Suppl. Fig. 4). We observed the opposite; therefore, higher values of *Ṡ_μ_*(*b* = 6000) and *f* are dominated by the greater density of neuronal and/or glial processes. In addition, we observed less alignment of these processes, *κ*. This particular combination supports the greater density of more orientationally-dispersed cellular processes, such as those arising from glial cells. Additionally, the increase in kurtosis and reduction in mobility in the extracellular space are both in agreement with a greater cellular density.

Based on these observations, we hypothesize that increased complexity in astrocytic processes may be contributing to the agedependency of the diffusion-weighted signal in typically developing children. A detectable change in astrocytes might be expected because of their abundance in the white matter [62], the size and complexity of their processes [74], and their critical role in protracted white matter development [84–86]. While oligodendrocytes have been well characterized in development and are known to be fundamental for myelin formation and maintenance [87–89], astrocytes also have many important roles, including providing trophic support of oligodendrocytes, formation of the blood brain barrier, synaptic pruning and neurotransmitter recycling [90]. Moreover, it has been demonstrated that astrocytes go through morphological and density changes during brain maturation [84, 91]. Diffusion-weighted MR spectroscopy might resolve the relative signal contributions of neuronal and glial cell types during development in the white matter [92]. It will be important in the future to conduct similar studies in larger samples, relating the microstructural findings to neurodevelopmental disorder and psychiatric risk - preferably using a longitudinal design to better understand these risks.

### Strengths and limitations

A distinguishing feature of the current study is the image contrast enhancement gained from using the unique ultra-strong gradients of the Connectom 3 T scanner. The strong gradients allow a given diffusion encoding to be achieved using a shorter echo time. In this way, diffusion scans were shortened significantly, thereby reducing T_2_-related signal loss, and boosting SNR by 40–70% in comparison with clinical MRI scanners. This increased the statistical power of the study to detect group differences; in part compensating for the low *N* associated with the 22q11.2DS participant group. In addition, recruiting children with 22q11.2DS enables us to investigate the neurodevelopmental pathways based on genotype, thus mitigating some of the difficulties with genetic and environmental heterogeneity in clinically ascertained groups such as ASD or schizophrenia. Overall, efforts to maximize contrast sensitivity of dMRI in both typically developing children and children with 22q11.2DS allowed for the investigation of microstructural hypo-connectivity in brain white matter.

Children with 22q11.2DS are harder to recruit than typically developing children due to the relative rarity of the syndrome and the frequency of MRI contraindications, e.g., history of cardiac surgery. This study was also severely challenged due to changing attitudes for in-person research visits during and post-COVID. However, despite the relatively low *N* compared to larger studies carried out across multiple sites, we observed similar effect sizes in diffusion metrics between 22q11.2DS and ASM controls. The enhanced sensitivity of our results was conferred by the strong gradients of the Siemens Connectom 3 T scanner, where high-*b* contrast acts as a filter for axons and glial processes, versus DTI (using low *b*) where microscopic differences are often masked. In addition, children with 22q11.2DS and the ASM group were within a narrow age-range (11 to 15), limiting the influence of variance due to known age-effects. Even so, the effect sizes reported for children with 22q11.2DS are still susceptible to magnitude and sign errors, and should be interpreted cautiously [93].

The current length of the image acquisition was demanding for children, particularly those with 22q11.2DS, although this was partly mitigated by providing the opportunity to acclimatize to the scanning procedure in a mock-scanner. Despite the richness of the protocol, certain recent developments in biophysical modeling, e.g., axon diameter mapping, could not be applied to these data, as the diffusion times were insufficiently short to resolve different diameters. As such, there was no direct in vivo quantification of axon diameter which would have further strengthened the validation of disrupted axon morphology (see Suppl. Fig. 4 on sensitivity to axon diameters). Despite earlier success in using the Connectom scanner to quantify axon diameters in vivo [30, 63, 94], these measurements require extensive scan times that are currently not compatible with the time constraints of scanning children. Characterizing axon diameter distributions in this cohort would either require a dedicated study, faster imaging techniques that maximize participant comfort, or the incorporation of motion correction strategies that do not rely on image-registration based approaches [95, 96]. It would also be interesting to include myelinsensitive contrasts, e.g. magnetization transfer imaging, as this information is orthogonal to dMRI and has been shown to be sensitive to both typical and atypical development [97, 98].

## Conclusions

In summary, our findings provide support that widespread differences in white matter volumes and microstructure are related to disruptions in axonal morphology in children with 22q11.2DS. Interestingly, the dMRI fingerprint of 22q11.2DS children was most similar in terms of biophysical principal components to those of older children from the TD cohort. Similar findings were also reported in previously published large-scale dMRI studies (i.e., low-*b* DTI). By exploiting the unique hardware used in this study, we extend the interpretation of dMRI signal beyond microstructural sensitivity towards cellular specificity. We demonstrate evidence for hypo-connectivity in 22q11.2DS, a hypothesis put forward in a preclinical setting, but for the first time observed in vivo. This is in contrast to new insights in TD, where glial processes are implicated in observed age-effects in white matter microstructure. The sensitivity and specificity provided by the combination of high-*b* dMRI and biophysical modelling highlights the discriminatory power of this approach for investigating dMRI signatures of both typical and atypical developmental populations.

## Supplementary Material

Supplementary Material

## Figures and Tables

**Table 1:** Demographics of typically developing (TD) and 22q11.2DS participants.

**TD participants**					
**Sex**	***N***	**Age**			
F	49	12.9 (3.1)			
M	43	11.8 (2.6)			
**Age- and sex-matched TD participants**					
**Sex**	***N***	**Age**	**IQ** ^*^		
F	14	12.9 (1.0)	101.8 (11.2)		
M	8	14.4 (0.8)	103.4 (11.1)		
**Participants with 22q11.2DS**					
**Sex**	***N***	**Age**	**IQ**	**SCQ**	**CAPA**
F	6	13.11 (0.87)	76.1 (8.8)	9.4 (4.4)	3.3 (4.7)
M	2	14.37 (1.16)	80.5 (27.6)	10.5 (4.9)	3.5 (0.7)

Mean and standard deviation are shown for all groups.

* Of the age- and sex-matched TD participants, two females and one male did not perform the abbreviated IQ test.

*M* male, *F* female, *N* number, *SCQ* Social Communication Questionnaire, *CAPA* Child and Adolescent Psychiatric Assessment.

**Table 2:** Standardized factor loadings for total white matter.

Metric	BPC1	BPC2	BPC3
FA	0.96	−0.13	−0.12
$\overset{-}{D}$	−0.10	0.97	0.09
*D*_⊥_	−0.30	0.87	0.09
*D*_∥_	0.26	1.02	0.01
$\overset{-}{K}$	0.02	0.06	0.99
*K*_⊥_	0.04	0.07	0.92
*K*_∥_	−0.04	−0.14	0.86
*Ṡ_μ_*(*b* = 500)	0.08	-0.85	0.25
*Ṡ_μ_*(*b* = 6000)	0.68	−0.11	0.41
*Ṡ_σ_*(*b* = 500)	1.00	0.13	−0.03
*Ṡ_σ_*(*b* = 6000)	0.79	−0.10	0.28

*FA* fractional anisotropy; $\overset{-}{D},$ mean diffusivity; *D*_⊥_, radial diffusivity; *D*_∥_, axial diffusivity; $\overset{-}{K},$ mean kurtosis; *K*_⊥_, radial kurtosis; *K*_∥_, axial kurtosis; *Ṡ_μ_*(*b* = 500), spherical mean at low-*b*; *Ṡ_μ_*(*b* = 6000), spherical mean at high-*b*; *Ṡ_σ_*(*b* = 500), spherical variance at low-*b*; *Ṡ_σ_*(*b* = 6000), spherical variance at high-*b*.

## Data Availability

Due to ethical concerns, supporting data cannot be made openly available. The computer code is available upon request from the first author.

## References

[1] Marshall CR, Howrigan DP, Merico D, Thiruvahindrapuram B, Wu W, Greer DS (2017). Contribution of copy number variants to schizophrenia from a genomewide study of 41,321 subjects. Nat Genet.

[2] McDonald-McGinn DM, Sullivan KE, Marino B, Philip N, Swillen A, Vorstman JAS (2015). 22Q11.2 Deletion Syndrome. Nat Rev Primer.

[3] Schneider M, Debbané M, Bassett AS, Chow EWC, Fung WLA, Van Den Bree MBM (2014). Psychiatric disorders from childhood to adulthood in 22q11.2 deletion syndrome: results from the international consortium on brain and behavior in 22q11. Am J Psych.

[4] Eaton CB, Thomas RH, Hamandi K, Payne GC, Kerr MP, Linden DEJ (2019). Epilepsy and seizures in young people with 22q11.2 deletion syndrome: prevalence and links with other neurodevelopmental disorders. Epilepsia.

[5] Cunningham AC, Delport S, Cumines W, Busse M, Linden DEJ, Hall J (2018). Developmental coordination disorder, psychopathology and IQ in 22q11.2 deletion syndrome. Br J Psychiatry.

[6] Moreau CA, Urchs SGW, Kuldeep K, Orban P, Schramm C, Dumas G (2020). Mutations associated with neuropsychiatric conditions delineate functional brain connectivity dimensions contributing to autism and schizophrenia. Nat Commun.

[7] Bartzokis G (2002). Schizophrenia: breakdown in the well-regulated lifelong process of brain development and maturation. Neuropsychopharmacology.

[8] Stephan KE, Friston KJ, Frith CD (2009). Dysconnection in schizophrenia: from abnormal synaptic plasticity to failures of self-monitoring. Schizophr Bull.

[9] Kelly S, Jahanshad N, Zalesky A, Kochunov P, Agartz I, Alloza C (2018). Widespread white matter microstructural differences in schizophrenia across 4322 individuals: Results from the ENIGMA Schizophrenia DTI Working Group. Mol Psych.

[10] Stessman HA, Bernier R, Eichler EE (2014). A genotype-first approach to defining the subtypes of a complex disease. Cell.

[11] Meechan DW, Maynard TM, Tucker ES, Fernandez A, Karpinski BA, Rothblat LA (2015). Modeling a model: Mouse genetics, 22q11.2 Deletion Syndrome, and disorders of cortical circuit development. Prog Neurobiol.

[12] Fernandez A, Meechan DW, Karpinski BA, Paronett EM, Bryan CA, Rutz HL (2019). Mitochondrial dysfunction leads to cortical under-connectivity and cognitive impairment. Neuron.

[13] Mukai J, Tamura M, Fénelon K, Rosen AM, Spellman TJ, Kang R (2015). Molecular substrates of altered axonal growth and brain connectivity in a mouse model of schizophrenia. Neuron.

[14] Sigurdsson T, Stark KL, Karayiorgou M, Gogos JA, Gordon JA (2010). Impaired hippocampal-prefrontal synchrony in a genetic mouse model of schizophrenia. Nature.

[15] Buzsaki G, Draguhn A (2004). Neuronal oscillations in cortical networks. Science.

[16] Perrin JS, Herve PY, Leonard G, Perron M, Pike GB, Pitiot A (2008). Growth of white matter in the adolescent brain: role of testosterone and androgen receptor. J Neurosci.

[17] Yakovlev PI, Lecours AR (1967). The myelogenetic cycles of regional maturation of the brain. Regional Development of Brain in Early Life.

[18] Einstein A (1905). Uber die von der molekularkinetischen Theorie der Warme geforderte Bewegumg von in ruhenden Flussigkeiten suspendierten Teilchen. Ann Phys.

[19] Le Bihan D, Breton E, Lallemand D, Grenier P, Cabanis E, Laval-Jeantet M (1986). MR imaging of intravoxel incoherent motions: application to diffusion and perfusion in neurologic disorders. Radiology.

[20] Beaulieu C (2002). The basis of anisotropic water diffusion in the nervous system-A technical review. NMR Biomed.

[21] Basser PJ, Pierpaoli C (1996). Microstructural and physiological features of tissues elucidated by quantitative-diffusion-tensor MRI. J Magn Reson-Ser B.

[22] Villalón-Reina JE, Martínez K, Qu X, Ching CRK, Nir TM, Kothapalli D (2020). Altered white matter microstructure in 22q11.2 deletion syndrome: a multisite diffusion tensor imaging study. Mol Psych.

[23] Lebel C, Beaulieu C (2011). Longitudinal development of human brain wiring continues from childhood into adulthood. J Neurosci.

[24] Stauffer EM, Bethlehem RAI, Warrier V, Murray GK, Romero-Garcia R, Seidlitz J (2021). Grey and white matter microstructure is associated with polygenic risk for schizophrenia. Mol Psych.

[25] Jones DK, Knösche TR, Turner R (2013). White matter integrity, fiber count, and other fallacies: the do’s and don’ts of diffusion MRI. NeuroImage.

[26] McNab JA, Edlow BL, Witzel T, Huang SY, Bhat H, Heberlein K (2013). The Human Connectome Project and beyond: initial applications of 300 mT/m gradients. NeuroImage.

[27] Jones DK, Alexander DC, Bowtell R, Cercignani M, Dell’Acqua F, McHugh DJ (2018). Microstructural imaging of the human brain with a ‘super-scanner’: 10 key advantages of ultra-strong gradients for diffusion MRI. NeuroImage.

[28] Veraart J, Fieremans E, Novikov DS (2019). On the scaling behavior of water diffusion in human brain white matter. NeuroImage.

[29] Novikov DS, Kiselev VG, Jespersen SN (2018). On modeling. Magn Reson Med.

[30] Veraart J, Nunes D, Rudrapatna U, Fieremans E, Jones DK, Novikov DS (2020). Noninvasive quantification of axon radii using diffusion MRI. eLife.

[31] Morrison S, Chawner SJRA, van Amelsvoort TAMJ, Swillen A, Vingerhoets C, Vergaelen E (2020). Cognitive deficits in childhood, adolescence and adulthood in 22q11.2 deletion syndrome and association with psychopathology. Transl Psychiatry.

[32] Wechsler D (1999). Wechsler Abbreviated Scale of Intelligence.

[33] Berument SK, Rutter M, Lord C, Pickles A, Bailey A (1999). Autism screening questionnaire: diagnostic validity. Br J Psychiatry.

[34] Angold A, Costello EJ, Messer SC, Pickles A, Winder F, Silver D (1995). Development of a short questionnaire for use in epidemiological studies of depression in children and adolescents. Int J Methods Psychiatr Res.

[35] Ottman R, Barker-Cummings C, Leibson CL, Vasoli VM, Hauser WA, Buchhalter JR (2010). Validation of a brief screening instrument for the ascertainment of epilepsy. Epilepsia.

[36] Jones DK, Simmons A, Williams SCR, Horsfield MA (1999). Non-invasive assessment of axonal fiber connectivity in the human brain via diffusion tensor MRI. Magn Reson Med.

[37] Veraart J, Fieremans E, Novikov DS (2016). Diffusion MRI noise mapping using random matrix theory. Magn Reson Med.

[38] Vos SB, Tax CMW, Luijten PR, Ourselin S, Leemans A, Froeling M (2017). The importance of correcting for signal drift in diffusion MRI. Magn Reson Med.

[39] Andersson JLR, Sotiropoulos SN (2016). An integrated approach to correction for off-resonance effects and subject movement in diffusion MR imaging. NeuroImage.

[40] Kellner E, Dhital B, Kiselev VG, Reisert M (2016). Gibbs-ringing artifact removal based on local subvoxel-shifts. Magn Reson Med.

[41] Sairanen V, Leemans A, Tax CMW (2018). Fast and accurate Slicewise OutLIer Detection (SOLID) with informed model estimation for diffusion MRI data. NeuroImage.

[42] Tournier JD, Smith R, Raffelt D, Tabbara R, Dhollander T, Pietsch M (2019). MRtrix3: A fast, flexible and open software framework for medical image processing and visualisation. NeuroImage.

[43] Jenkinson M, Beckmann CF, Behrens TEJ, Woolrich MW, Smith SM (2012). FSL. NeuroImage.

[44] Chamberland M, Raven EP, Genc S, Duffy K, Descoteaux M, Parker GD (2019). Dimensionality reduction of diffusion MRI measures for improved tractometry of the human brain. NeuroImage.

[45] Zhang Y, Brady M, Smith S (2001). Segmentation of brain MR images through a hidden Markov random field model and the expectation-maximization algorithm. IEEE Trans Med Imaging.

[46] Wasserthal J, Neher PF, Hirjak D, Maier-Hein KH (2019). Combined tract segmentation and orientation mapping for bundle-specific tractography. Med Image Anal.

[47] Calamante F, Tournier JD, Jackson GD, Connelly A (2010). Track-density imaging (TDI): super-resolution white matter imaging using whole-brain track-density mapping. NeuroImage.

[48] Jelescu IO, Veraart J, Fieremans E, Novikov DS (2016). Degeneracy in model parameter estimation for multi-compartmental diffusion in neuronal tissue. NMR Biomed.

[49] Klein J, Hermann S, Konrad O, Hahn HK, Peitgen HO (2007). Automatic quantification of DTI parameters along fiber bundles. Bildverarbeitung für die Medizin.

[50] Yeatman JD, Dougherty RF, Myall NJ, Wandell BA, Feldman HM (2012). Tract profiles of white matter properties: automating fiber-tract quantification. PLoS ONE.

[51] Maier-Hein KH, Neher PF, Houde JC, Côté MA, Garyfallidis E, Zhong J (2017). The challenge of mapping the human connectome based on diffusion tractography. Nat Commun.

[52] Benjamini Y, Hochberg Y (1995). Controlling the false discovery rate: a practical and powerful approach to multiple testing. J R Stat Soc.

[53] Lakens D (2013). Calculating and reporting effect sizes to facilitate cumulative science: a practical primer for t-tests and ANOVAs. Front Psychol.

[54] R Core Team (2020). R: A language and environment for statistical computing.

[55] Shi J, Yang S, Wang J, Huang S, Yao Y, Zhang S (2019). Detecting normal pediatric brain development with diffusional kurtosis imaging. Eur J Radio.

[56] Giedd JN, Blumenthal J, Jeffries NO, Castellanos FX, Liu H, Zijdenbos A (1999). Brain development during childhood and adolescence: a longitudinal MRI study. Nat Neurosci.

[57] Durston S, Pol HEH, Casey B, Giedd JN, Buitelaar JK, van Engeland H (2001). Anatomical MRI of the developing human brain: what have we learned?. J Am Acad Child Adolesc Psych.

[58] de Mooij SMM, Henson RNA, Waldorp LJ, Kievit RA (2018). Age differentiation within gray matter, white matter, and between memory and white matter in an adult life span cohort. J Neurosci.

[59] Cox SR, Ritchie SJ, Tucker-Drob EM, Liewald DC, Hagenaars SP, Davies G (2016). Ageing and brain white matter structure in 3,513 UK Biobank participants. Nat Commun.

[60] Rogdaki M, Gudbrandsen M, McCutcheon RA, Blackmore CE, Brugger S, Ecker C (2020). Magnitude and heterogeneity of brain structural abnormalities in 22q11.2 deletion syndrome: a meta-analysis. Mol Psych.

[61] Stuermer CAO (1984). Rules for retinotectal terminal arborizations in the goldfish optic tectum: a whole-mount study. J Comp Neurol.

[62] Perge JA, Koch K, Miller R, Sterling P, Balasubramanian V (2009). How the optic nerve allocates space, energy capacity, and information. J Neurosci.

[63] Drakesmith M, Harms R, Rudrapatna SU, Parker GD, Evans CJ, Jones DK (2019). Estimating axon conduction velocity in vivo from microstructural MRI. NeuroImage.

[64] Judson MC, Burette AC, Thaxton CL, Pribisko AL, Shen MD, Rumple AM (2017). Decreased axon caliber underlies loss of fiber tract integrity, disproportional reductions in white matter volume, and microcephaly in Angelman syndrome model mice. J Neurosci.

[65] Wegiel J, Kaczmarski W, Flory M, Martinez-Cerdeno V, Wisniewski T, Nowicki K (2018). Deficit of corpus callosum axons, reduced axon diameter and decreased area are markers of abnormal development of interhemispheric connections in autistic subjects. Acta Neuropathol Commun.

[66] Zikopoulos B, Barbas H (2010). Changes in prefrontal axons may disrupt the network in autism. J Neurosci.

[67] Konopaske GT, Lange N, Coyle JT, Benes FM (2014). Prefrontal cortical dendritic spine pathology in schizophrenia and bipolar disorder. JAMA Psych.

[68] Nehme R, Pietiläinen O, Artomov M, Tegtmeyer M, Valakh V, Lehtonen L (2022). The 22q11.2 region regulates presynaptic gene-products linked to schizophrenia. Nat Commun.

[69] Fiksinski AM, Hoftman GD, Vorstman JAS, Bearden CE (2023). A genetics-first approach to understanding autism and schizophrenia spectrum disorders: the 22q11.2 deletion syndrome. Mol Psychiatry.

[70] Burcaw LM, Fieremans E, Novikov DS (2015). Mesoscopic structure of neuronal tracts from time-dependent diffusion. NeuroImage.

[71] Takahashi M, Hackney DB, Zhang G, Wehrli SL, Wright AC, O’Brien WT (2002). Magnetic resonance microimaging of intraaxonal water diffusion in live excised lamprey spinal cord. Proc Natl Acad Sci.

[72] Lamantia AS, Rakic P (1990). Cytological and quantitative characteristics of four cerebral commissures in the rhesus monkey. J Comp Neurol.

[73] Barres BA (2008). The mystery and magic of glia: a perspective on their roles in health and disease. Neuron.

[74] Oberheim NA, Takano T, Han X, He W, Lin JHC, Wang F (2009). Uniquely hominid features of adult human astrocytes. J Neurosci.

[75] Schwartz ED, Cooper ET, Fan Y, Jawad AF, Chin CL, Nissanov J (2005). MRI diffusion coefficients in spinal cord correlate with axon morphometry. NeuroReport.

[76] Takahashi M, Ono J, Harada K, Maeda M, Hackney DB (2000). Diffusional anisotropy in cranial nerves with maturation: quantitative evaluation with diffusion MR imaging in rats. Radiology.

[77] Paus T (2010). Growth of white matter in the adolescent brain: myelin or axon?. Brain Cogn.

[78] Hagmann P, Sporns O, Madan N, Cammoun L, Pienaar R, Wedeen VJ (2010). White matter maturation reshapes structural connectivity in the late developing human brain. Proc Natl Acad Sci.

[79] Genc S, Tax CMW, Raven EP, Chamberland M, Parker GD, Jones DK (2020). Impact of b-value on estimates of apparent fibre density. Hum Brain Mapp.

[80] Almeida RG, Lyons DA (2017). On myelinated axon plasticity and neuronal circuit formation and function. J Neurosci.

[81] Costa AR, Pinto-Costa R, Sousa SC, Sousa MM (2018). The regulation of axon diameter: from axonal circumferential contractility to activity-dependent axon swelling. Front Mol Neurosci.

[82] Chowen JA, Azcoitia I, Cardona-Gomez GP, Garcia-Segura LM (2000). Sex steroids and the brain: lessons from animal studies. J Pediatr Endocrinol Metab.

[83] Maninger N, Wolkowitz OM, Reus VI, Epel ES, Mellon SH (2009). Neurobiological and neuropsychiatric effects of dehydroepiandrosterone (DHEA) and DHEA sulfate (DHEAS). Front Neuroendocrinol.

[84] Robillard KN, Lee KM, Chiu KB, MacLean AG (2016). Glial cell morphological and density changes through the lifespan of rhesus macaques. Brain Behav Immun.

[85] Sigaard RK, Kjær M, Pakkenberg B (2016). Development of the cell population in the brain white matter of young children. Cereb Cortex.

[86] Yoon H, Walters G, Paulsen AR, Scarisbrick IA (2017). Astrocyte heterogeneity across the brain and spinal cord occurs developmentally, in adulthood and in response to demyelination. PLoS ONE.

[87] Hildebrand C, Remahl S, Persson H, Bjartmar C (1993). Myelinated nerve fibres in the CNS. Prog Neurobiol.

[88] Bartzokis G (2012). Neuroglialpharmacology: myelination as a shared mechanism of action of psychotropic treatments. Neuropharmacology.

[89] Fields RD (2008). White matter in learning, cognition and psychiatric disorders. Trends Neurosci.

[90] Verkhratsky A, Nedergaard M (2018). Physiology of astroglia. Physiol Rev.

[91] Molofsk AV, Krenick R, Ullian E, Tsai HH, Deneen B, Richardson WD (2012). Astrocytes and disease: a neurodevelopmental perspective. Genes Dev.

[92] Ligneul C, Palombo M, Hernández-Garzón E, Carrillo-de Sauvage MA, Flament J, Hantraye P (2019). Diffusion-weighted magnetic resonance spectroscopy enables cell-specific monitoring of astrocyte reactivity in vivo. NeuroImage.

[93] Gelman A, Carlin J (2014). Beyond power calculations: assessing Type S (Sign) and Type M (Magnitude) errors. Perspect Psychol Sci.

[94] Huang SY, Tian Q, Fan Q, Witzel T, Wichtmann B, McNab JA (2020). High-gradient diffusion MRI reveals distinct estimates of axon diameter index within different white matter tracts in the in vivo human brain. Brain Struct Funct.

[95] Callaghan MF, Josephs O, Herbst M, Zaitsev M, Todd N, Weiskopf N (2015). An evaluation of prospective motion correction (PMC) for high resolution quantitative MRI. Front Neurosci.

[96] Herbst M, Zahneisen B, Knowles B, Zaitsev M, Ernst T (2015). Prospective motion correction of segmented diffusion weighted EPI. Magn Reson Med.

[97] Whitaker KJ, Vértes PE, Romero-Garcia R, Váša F, Moutoussis M, Prabhu G (2016). Adolescence is associated with genomically patterned consolidation of the hubs of the human brain connectome. Proc Natl Acad Sci.

[98] Romero-Garcia R, Seidlitz J, Whitaker KJ, Morgan SE, Fonagy P, Dolan RJ (2020). Schizotypy-related magnetization of cortex in healthy adolescence Is colocated with expression of schizophrenia-related genes. Biol Psych.

