# Supplementary material for: In vivo evidence of microstructural hypo-connectivity of brain white matter in 22q11.2 deletion syndrome
Source: Mol Psychiatry. Author manuscript; Available in PMC 2024 Jan 30. (PMC7615578; doi:10.1038/s41380-023-02178-w)
Supplement: Supplementary Material [file EMS189818-supplement-Supplementary_Material.pdf]

Descriptions of diffusion metrics

- *Diffusion tensor and kurtosis imaging:* Diffusion tensor (DT-MRI) (1) and diffusion kurtosis imaging (DK-MRI) (2) have been used extensively in studies of white matter microstructure in typical development (see review, (3)) and children with 22q11.2DS (4). DT-MRI metrics include fractional anisotropy (FA), mean diffusivity ($\overline{D}$), radial diffusivity ($D_{\perp}$) and axial diffusivity ($D_{\parallel}$). DK-MRI metrics include mean kurtosis ($\overline{K}$), radial kurtosis ($K_{\perp}$), and axial kurtosis ($K_{\parallel}$), and are shown to be intrinsically more sensitive to compartmental microstructural attributes than DT-MRI parameters (see review, (5)). The diffusion and kurtosis tensors were estimated from the data with $b \leq 2400 s/mm^2$ (6). To improve the robustness of these parameters, prior to fitting the kurtosis tensor, the data were first smoothed with an anisotropic Gaussian filter (7) that was aligned with the underlying fiber architecture.
- *Spherical moments:* By increasing diffusion weightings, signal is suppressed from the extracellular matrix thereby allowing for more direct sampling of intracellular features at higher b-values. This is a unique feature of this study in terms of b-values that can be achieved on the Connectom scanner. Then, without imposing any biophysical model or even prior knowledge, we were able to compute the spherical *mean* and spherical *variance* of the diffusion-weighted MRI signal at each distinct diffusion weighting, b = 500, 1200, 2400, 4000, and $6000 s/mm^2$ using the power of the 0th and 2nd order spherical harmonic coefficients (8). At high b-values, e.g. $b = 6000 s/mm^2$, the spherical mean $\dot{S}_{\mu}(b)$ and spherical variance $\dot{S}_{\sigma}(b)$ relate strongly with specific information about the intra-cellular microstructure, including the axon diameter and the intracellular signal

fraction and diffusivity (See Suppl. Section *Sensitivity to the axon morphology*) (9). Unlike $\dot{S}_\mu(b)$, $\dot{S}_\sigma(b)$ also encodes the orientational dispersion (10).

- *Biophysical Standard Model:* The richness of the diffusion-weighted data acquisition enabled us to adopt a minimally constrained biophysical model, thereby minimizing previously reported biases arising from unvalidated or contested assumptions (10). Here, we adopt the Biophysical Standard Model, or BSM, of white matter to extract more specific biophysical features. The model describes restricted diffusion within dispersed neuronal and/or glial processes, embedded in an effective extra-cellular matrix (10). The model is parameterized by intracellular signal fraction (*f*), parallel intracellular diffusivity (D_c), parallel and perpendicular extracellular diffusivity ($D_e^{\parallel}$, $D_e^{\perp}$) and orientational alignment (κ). Note 0 ≤ κ ≤ 1 with $\boldsymbol{\kappa}$ = 0 for fully isotropic diffusion cf. p_2 in Novikov et al (10).

Precision of diffusion metrics The precision of the individual diffusion metrics was evaluated using a Monte-Carlo simulation with 100,000 trials in which noise was added to synthetic data. The synthetic data were generated using the biophysical Standard Model (BSM) with realistic ground truth parameters, based on our data, while mimicking the acquisition settings of the study. The noise level was chosen to match the signal-to-noise ratio of the data itself. For each noise realization, all diffusion metrics were estimated and the variability across the different realizations are shown in Suppl. Fig 2. The coefficients of variation are 0.53, 0.82, 1.68, 1.09, 2.88, 3.42, 7.07, 0.41, 1.73, 1.58, 2.10, 2.93, .330, 8.68, 6.07, and 1.99% for FA, $\dot{S}_\mu(b = 6000)$, $\dot{S}_\sigma(b = 500)$, $\dot{S}_\sigma(b = 6000)$, $\overline{D}$, $D_\perp$, $D_\parallel$, $\dot{S}_\mu(b = 500)$, $\overline{K}$, $K_\perp$, $K_\parallel$, *f*, D_c, $D_e^{\parallel}$, $D_e^{\perp}$, and κ, respectively. The kurtosis metrics and, particularly, the compartmental diffusivities of BSM have poor

performance in terms of precision. Therefore, we applied smoothing and along-tract analysis to improve the precision of DKI and BSM. However, the sensitivity of the compartmental diffusivities D_c, $D_e^{\parallel}$, $D_e^{\perp}$, is intrinsically challenged by the low precision of the estimator.

Tract Volume Quantification To evaluate the reproducibility of the tract volume quantification, we re-analyzed test/retest data of five healthy volunteers that were collected during two scanning sessions with exactly the same imaging protocol on the Siemens Connectom 3T MR scanner. For each volunteer, the two test-retest scanning sessions were performed on the same day interleaved by a short break. In both sessions, subjects were re-positioned by the same operator. Despite some minor changes in imaging protocol in comparison to the study-specific data, fiber tracking and automated segmentation was performed on diffusion-weighted images with b = 6000s/mm^2.

An overview of the most relevant scan parameters: TR/TE = 3500/66ms, imaging matrix: 88 × 88, voxel dimensions: 2.5 × 2.5 × 2.5mm^3, scan acceleration using SMS=2 and GRAPPA (R=2).

The inter- and intra-subject variability of the estimated volume of all eleven tracts was computed. The variability served as an input to compute the minimally required effect size that could be detected with statistical significance in our study. For this power analysis, we used *G*Power*. In Suppl. Fig. 3, we show the minimally required effect size as a function of the tract volume for each tract. The technique itself lacks sensitivity to detect subtle effects, but is useful for highlighting changes in tract volume of more than 10-20%.

Sensitivity to the axon morphology Even when deploying the exceptionally strong gradients of the Siemens Connectom 3T scanner, the diffusion-weighted signal attenuation perpendicular

to a micrometer-thin axons is weak (11). Aside from the low sensitivity, our study lacks data with changing diffusion time and/or additional ultra-high *b*-values to enable the quantification of the axon diameter directly (9). Nonetheless, various diffusion parameters have previously been shown to correlate with the axon diameter, e.g. $D_{\perp}$ (12,13).

In our study, we maximized the direct sensitivity of dMRI to differences in axon diameter by evaluating the spherical mean at a high *b*-value, while minimizing the gradient duration δ = 7ms and diffusion time Δ = 23.3ms. As a result, $\dot{S}_{\mu}(b = 6000)$ correlates strongly with $\frac{\mathrm{f}}{D_c} S_{\perp}^{\mathrm{Axon}}(r)$, with $S_{\perp}^{\mathrm{Axon}}(r)$ the radial signal attenuation inside a cylindrical axon with radius *r*. In van Gelderen et al (11), it was demonstrated that,

$$\ln S_{\perp}^{\mathrm{Axon}}(r) = -\frac{2q^2r^4}{D_0}\sum_{m=1}^{\infty}\frac{t_c}{\alpha_m^6(\alpha_m^2-1)}\cdot\left[2\alpha_m^2\frac{\delta}{t_c} - 2 + 2e^{-\alpha_m^2\delta/t_c} + 2e^{-\alpha_m^2\Delta/t_c} - e^{-\alpha_m^2(\Delta-\delta)/t_c} - e^{-\alpha_m^2(\Delta+\delta)/t_c}\right] + \mathcal{O}(q^4)$$

where *q* = γG is the diffusion-weighting wave vector with γ the gyromagnetic ratio for protons and *G* the gradient strength. Furthermore, D_0 is the diffusivity of the axoplasm, α_m is the mth root of $\mathrm{d}J_1(\alpha)/\mathrm{d}\alpha = 0$, and $J_1(\alpha)$ is the Bessel function of the first kind. Here, $t_c = r^2/D_0$ is the diffusion time across the axon; see Suppl. Fig. 4.

The reduction of axon diameter specifically will directly result in a higher $\dot{S}_{\mu}(b = 6000)$. This effect is mainly prominent if large axons are affected, but weakens significantly at lower *b*-values, in part because the extra-cellular signal is not fully-suppressed and is likely to mask the effects at *b* < 6000s/mm^2 (14). The unique gradient strength of the scanner used here was critical to our exploration of the axonal morphology, because a similar sensitivity cannot be achieved on clinical MR scanners with gradient strengths up to 80mT/m, even for the same *b*-values, Suppl. Fig. 4.

The biophysical interpretation of the BPC Here we provide a more in-depth justification for our biophysical interpretation of the first BPC: intracellular signal.

The first BPC is comprised of FA, $\dot{S}_\sigma$ (low and high *b*), and $\dot{S}_\mu$ (high *b* only). On its own, FA is not specific to any particular microstructural property, e.g., it might reflect differences in orientational dispersion or cellular packing, or both, and this has been the Achilles heel of diffusion MR since its inception decades ago (15–18).

Evidence of strong co-variance with more specific and interpretable metrics, such as $\dot{S}_\sigma$ and $\dot{S}_\mu$ obtained at high *b*-values, allows for a more specific interpretation. As high *b*-value diffusion-weighting suppresses the signal from the extracellular matrix (on account of the higher mobility in this space), the specificity to the intracellular (i.e., neuronal or glial) signal fraction increases (14).

At high *b*-values, both $\dot{S}_\sigma$ and $\dot{S}_\mu$ are sensitive to the intracellular signal fraction, whereas only $\dot{S}_\sigma$ is sensitive to orientational dispersion. Therefore, if orientational dispersion had been a dominant biophysical feature, then $\dot{S}_\sigma$ would have reflected this, and $\dot{S}_\sigma$ and $\dot{S}_\mu$ would not have loaded onto the same representative component. The fact that these measures do, indeed, covary so strongly allows us to conclude that it is changes in the intracellular signal fraction that drive age-dependent increases in FA, rather than changes in orientational alignment - a process typically lumped into descriptions of FA. Furthermore, this finding is reinforced by concomitant higher values in BSM parameter *f*, the intracellular signal fraction.

Comparison to ENIGMA DT-MRI results The large-scale ENIGMA-22q11.2DS DT-MRI data set is an important benchmark and can be used as a reference point to the DT-MRI results of this study (4). Overall, a wide-spread WM phenotype of higher FA in 22q11.2DS versus lower

diffusivities (mean diffusivity ($\overline{D}$), radial diffusivity ($D_{\perp}$), and axial diffusivity ($D_{\parallel}$)) is reflected across both studies. However, the ENIGMA group found an inverse direction for the association tract, SLF (low FA relative to controls). This, alongside all comparable results, are presented in the table below. Nonsignificant results were not evaluated, as these lack statistical inference to prove the null hypothesis (i.e., no effect). The best match possible from the ROI-based technique is presented alongside the tracts included from this study.

The approaches used by each study are well accepted within the dMRI field. However, there are many techniques available, which have unknown contributions from both biological and nonbiological effects (for recent evaluation, see (19)).

The ENIGMA study segments WM by aligning individual datasets to an atlas of WM regions of interest. The atlas labels consist of discrete regions or segments from a particular pathway and do not directly correspond to the extent or shape of an individually defined WM pathway. In this study, we apply tractography to each individual dataset. When comparing ROIs to tracts, we find that there are notable differences in segmentation, even when sharing the same label. The extent of these differences varies depending on the tract or region being evaluated, posing an even greater challenge to harmonize results between studies.

SLF (FA result): The SLF is a large white matter bundle composed of three, functionally distinct fascicles. Three fascicles are provided by TractSeg (SLF-I, SLF-II, SLF-III), but SLF-II was selected as it is considered the largest, primary component of the tract. When compared to JHU-atlas, the ROI labeled 'SLF' was most representative of the tract SLF-III and inferior portions of SLF-II. There were no alternative ROIs to capture more superior aspects of SLF. As such, it is not possible to make a direct comparison; see Suppl. Fig. 5.

A recent DTI study with 101 participants with 22q11.2DS and 100 controls used a different tractography approach to define the SLF as two distinct branches (20,21). They report

increased FA for the superior SLF-parietal branch, but not inferior SLF-temporal branch. The SLF-temporal tract is most similar to SLF-III (TractSeg) and the SLF-ROI (ENIGMA).

Supplemental References

1. Basser PJ, Pierpaoli C. Microstructural and physiological features of tissues elucidated by quantitative-diffusion-tensor MRI. J Magn Reson - Ser B. 1996;111(3):209–19.

2. Jensen JH, Helpern JA, Ramani A, Lu H, Kaczynski K. Diffusional Kurtosis Imaging: the quantification of non-Gaussian water diffusion by means of magnetic resonance imaging. Magn Reson Med. 2005;53(6):1432–40.

3. Lebel C, Treit S, Beaulieu C. A review of diffusion MRI of typical white matter development from early childhood to young adulthood. NMR Biomed. 2017;(October 2016):1–23.

4. Villalón-Reina JE, Martínez K, Qu X, Ching CRK, Nir TM, Kothapalli D, et al. Altered white matter microstructure in 22q11.2 deletion syndrome: a multisite diffusion tensor imaging study. Mol Psychiatry. 2020 Nov 1;25(11):2818–31.

5. Jensen JH, Helpern JA. MRI quantification of non-Gaussian water diffusion by kurtosis analysis. NMR Biomed. 2010;23(7):698–710.

6. Veraart J, Sijbers J, Sunaert S, Leemans A, Jeurissen B. Weighted linear least squares estimation of diffusion MRI parameters: strengths, limitations, and pitfalls. NeuroImage. 2013;81:335–46.

7. Parker GJM, Schnabel JA, Symms MR, Werring DJ, Barker GJ. Nonlinear smoothing for reduction of systematic and random errors in diffusion tensor imaging. J Magn Reson Imaging. 2000;11(6):702–10.

8. Mirzaalian H, Ning L, Savadjiev P, Pasternak O, Bouix S, Michailovich O, et al. Inter-site and inter-scanner diffusion MRI data harmonization. NeuroImage. 2016;135:311–23.

9. Veraart J, Nunes D, Rudrapatna U, Fieremans E, Jones DK, Novikov DS, et al. Noninvasive quantification of axon radii using diffusion MRI. eLife. 2020;9:1–27.

10. Novikov DS, Veraart J, Jelescu IO, Fieremans E. Rotationally-invariant mapping of scalar and orientational metrics of neuronal microstructure with diffusion MRI. NeuroImage. 2018;174:518–38.

11. van Gelderen P, DesPres D, van Zijl P, Moonen C. Evaluation of restricted diffusion in cylinders. Phosphocreatine in rabbit leg muscle. J Magn Reson. 1994;103:255–60.

12. Takahashi M, Hackney DB, Zhang G, Wehrli SL, Wright AC, O'Brien WT, et al. Magnetic resonance microimaging of intraaxonal water diffusion in live excised lamprey spinal cord. Proc Natl Acad Sci. 2002;99(25).

13. Barazany D, Basser PJ, Assaf Y. In vivo measurement of axon diameter distribution in the corpus callosum of rat brain. Brain. 2009 May;132(Pt 5):1210–20.

14. Veraart J, Fieremans E, Novikov DS. On the scaling behavior of water diffusion in human brain white matter. NeuroImage. 2019;185:379–87.

15. Le Bihan D, Breton E, Lallemand D, Grenier P, Cabanis E, Laval-Jeantet M. MR imaging of intravoxel incoherent motions: application to diffusion and perfusion in neurologic disorders. Radiology. 1986;161:401–7.

16. Beaulieu C. The basis of anisotropic water diffusion in the nervous system - A technical review. NMR Biomed. 2002;15(7–8):435–55.

17. Jones DK, Knösche TR, Turner R. White matter integrity, fiber count, and other fallacies: the do's and don'ts of diffusion MRI. NeuroImage. 2013 Jun;73:239–54.

18. Tanner J. Self diffusion of water in frog muscle. Biophys J. 1979;28(1):107–16.

19. Schilling KG, Rheault F, Petit L, Hansen CB, Nath V, Yeh FC, et al. Tractography dissection variability: What happens when 42 groups dissect 14 white matter bundles on the same dataset? NeuroImage. 2021 Nov 1;243:118502.

20. Bagautdinova J, Padula MC, Zöller D, Sandini C, Schneider M, Schaer M, et al. Identifying neurodevelopmental anomalies of white matter microstructure associated with high risk for psychosis in 22q11.2DS. Transl Psychiatry. 2020 Nov 24;10(1):408.

21. Warrington S, Bryant KL, Khrapitchev AA, Sallet J, Charquero-Ballester M, Douaud G, et al. XTRACT - Standardised protocols for automated tractography in the human and macaque brain. NeuroImage. 2020;217(May):1–15.

Supplemental Figures

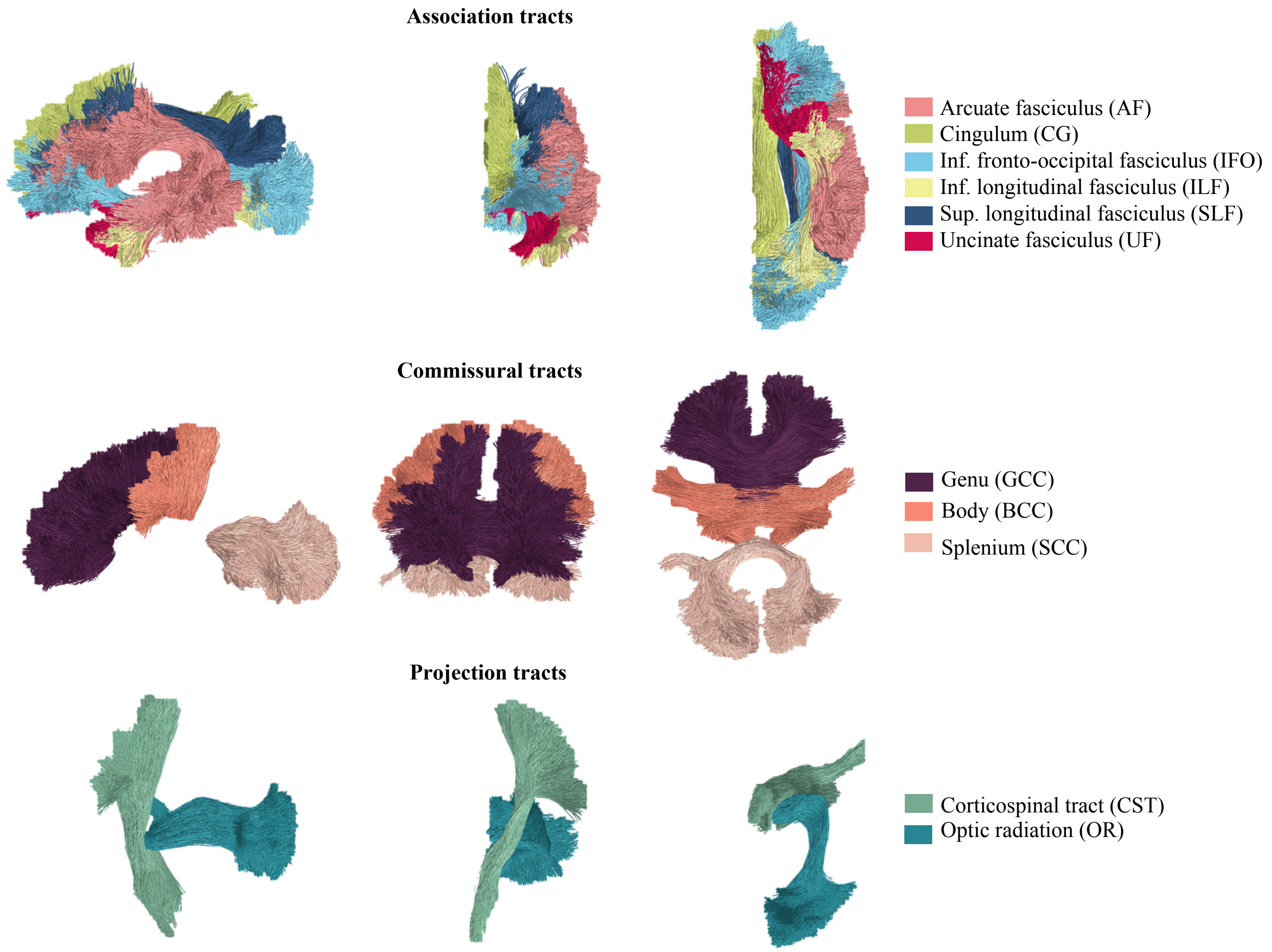

Suppl. Figure 1: White matter tracts of interest. Eleven fiber tracts extracted using automated segmentation software are shown for an 8-year-old female TD participant. Tracts are grouped by association (top), commissural (middle), and projection (bottom) pathways and shown for left hemisphere.

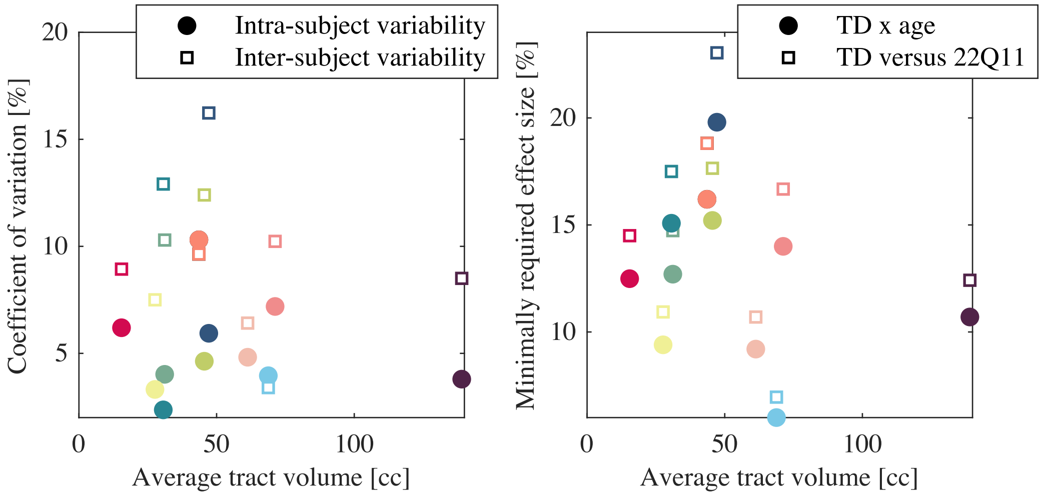

Suppl. Figure 2: Reproducibility of tract volume quantification. A) The coefficient of variation is shown for intra-subject (closed circle) and inter-subject (open square) tract volume measurements. B) The minimally required effect size is shown for detecting effects in TD (closed circle) and in 22q11.2DS (open square).

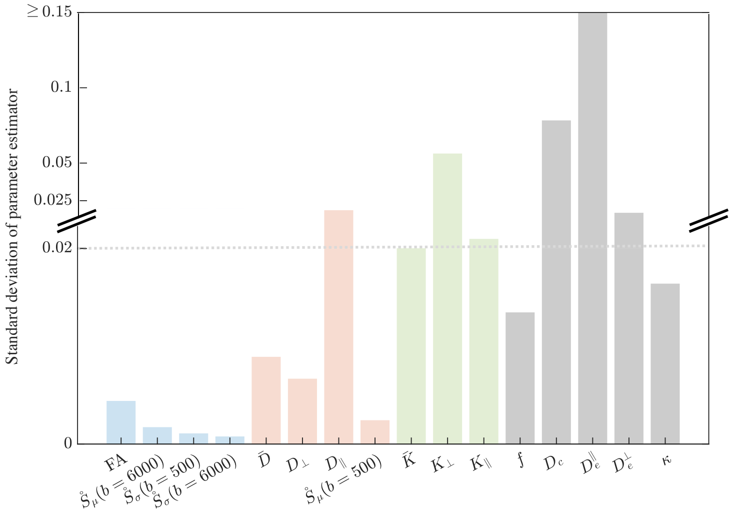

Suppl. Figure 3: The bar plot shows the standard deviation of the estimator of the various diffusion metrics, as predicted from the simulation experiment.

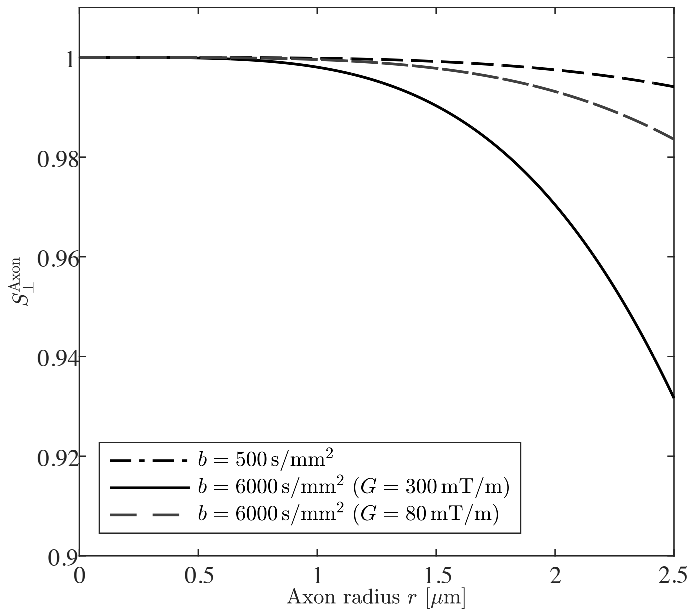

Suppl. Figure 4: Radial signal decay $S_\perp^{\text{Axon}}$ inside a cylindrical axon with radius *r*. The signal decay is evaluated for various *b*-values and gradient strengths to demonstrate the feasibility of the study scanner, i.e. Siemens Connectom 3T with *G* = 300mT/m, to sense axon calibers at high *b*-values and compare its performance to state-of-the-art clinical scanners with *G* = 80mT/m.

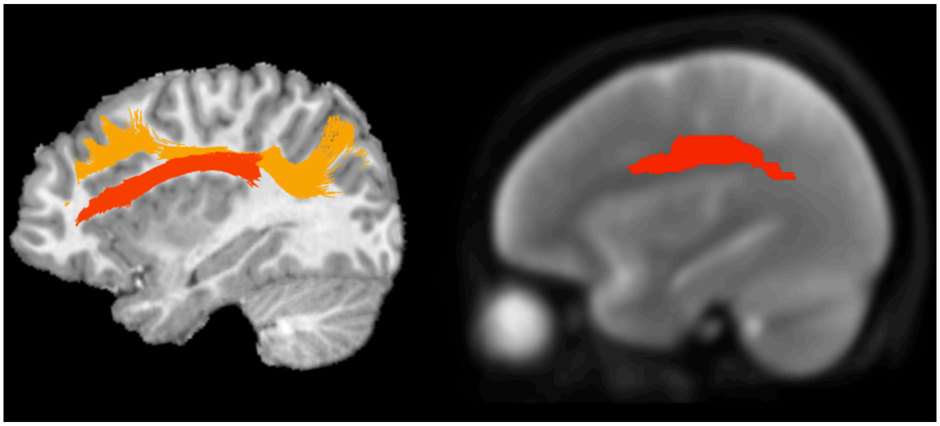

Suppl. Figure 5: Sagittal view of SLF. (left) TractSeg SLF II-tract (orange) and SLF III-tract (red) overlaid on an individual subject. (right) ICBM JHU WM atlas SLF-ROI overlaid on the T2 template.
