# Supplementary material for: In vivo evidence of microstructural hypo-connectivity of brain white matter in 22q11.2 deletion syndrome
Source: Mol Psychiatry. 2023 Jul 26;28(10):4342–52. doi: 10.1038/s41380-023-02178-w (PMC7615578; doi:10.1038/s41380-023-02178-w)
Supplement: Supplementary file 2 [file 41380_2023_2178_MOESM2_ESM.pdf]

Supplementary Table 1

Tractography (current study)						JHU Atlas (ENIGMA)					
**Tract**	**Label**	**FA**	**MD**	**AD**	**RD**	**Region of Interest**	**Label**	**FA**	**MD**	**AD**	**RD**
**Association Tracts**						**Association ROIs**					
Arcuate Fasiculus	AF	↑	↓	↓ **(L)**	↓	Anterior corona radiata	ACR	↑	↓	↓	↓
Cingulum	CG	↑	↓	↓	↓	Cingulum	CGC	↑	↓	↓	↓
Inf. fronto-occipital fasiculus*	IFO	↑	↓	ns	↓	Inf. fronto-occipital fasiculus	IFO	-	-	-	-
						Sagittal stratum	SS	ns	↓	↓	↓
						Anterior corona radiata	ACR	↑	↓	↓	↓
Inf. longitudinal fasiculus	ILF	↑ (R)	ns	ns	↓	Sagittal stratum	SS	ns	↓	↓	↓
Sup. longitudinal fasiculus II	SLF	↑	↓	↓ **(L)**	↓	Superior longitudinal fasciculus	SLF	↓	↓	↓	↓
Uncinate fasciculus	UF	ns	↓	↓ **(R)**	ns	Uncinate fasciculus	UNC	ns	↓	↓	ns
**Commissural Tracts**						**Commissural ROIs**					
Genu of Corpus Callosum	GCC	↑	↓	↓	↓	Genu of Corpus Callosum	GCC	↑	↓	↓	↓
Body of Corpus Callosum	BCC	↑	↓	ns	↓	Body of Corpus Callosum	BCC	↑	↓	↓	↓
Splenium of Corpus Callosum	SCC	ns	ns	ns	↓	Splenium of Corpus Callosum	SCC	↑	↓	↓	↓
**Projection Tracts**						**Projection ROIs**					
Corticospinal Tract*	CST	↑ (R)	↓ (R)	ns	↓	Superior corona radiata	SCR	↑	↓	↓	↓
						Posterior limb of internal capsule	PLIC	↑	↓	↑	↓
						Cerebral peduncle	CP	-	-	-	-
						Corticospinal tract	CST	x	x	x	x
Optic Radiation	OR	ns	↓	ns	↓	Posterior Thalamic Radiation	PTR	ns	↓	↓	↓

Suppl. Table 1. The current study separated tracts by left and right side. If an effect was present on one side only, it is noted with a (L) or (R). For ROIs that were not available, cells use (-); for the CST-ROI that was excluded, cells use (x). Nonsignificant results are 'ns'. *** A direct comparison is not feasible due to the many to one mapping of ROIs to tracts and incomplete data.
